# Polytopal Rearrangement Governing Stereochemistry of Bicyclic Oxime Ether Synthesis

**DOI:** 10.3390/ijms232012331

**Published:** 2022-10-15

**Authors:** Zlatan Spahić, Tomica Hrenar, Ines Primožič

**Affiliations:** Department of Chemistry, Faculty of Science, University of Zagreb, Horvatovac 102A, 10000 Zagreb, Croatia

**Keywords:** *O*-substituted ketoximes, stereoselectivity, mechanochemical and microwave synthesis, conformational analysis

## Abstract

In the present study, four *O*-substituted oximes of quinuclidin-3-one were synthesized using appropriate *O*-substituted hydroxylamine hydrochlorides. In order to perform these reactions in a solvent, a mixture of (*E*) and (*Z*) products was yielded. Using mechanochemical and microwave synthesis, we then obtained pure (*E*) oximes. In almost all cases, the conversion to oxime ethers was completed. Reactions were monitored by ATR spectroscopy and the ratios of (*E*) and (*Z*) oxime ethers were deduced from ^1^H NMR data. Several reactions were very rapid (1 min) with 100% conversion and stereospecificity. To investigate the reaction mechanisms, full conformational analyses of the reaction intermediates were performed and the lowest energy conformers were determined. These conformers differed in spatial arrangement around the nitrogen atom of the amino group and were in the correct orientation for reactions to occur. Calculated standard Gibbs energies of the formation were in agreement with the experimentally obtained ratios of (*E)* and (*Z*) isomers. This work shows alternatives to the classical synthesis of *O*-substituted oxime ether precursors and highlights the fast reaction rate and stereoselectivity of microwave synthesis as well as the “green” aspects of mechanochemistry.

## 1. Introduction

**Figure 1:**
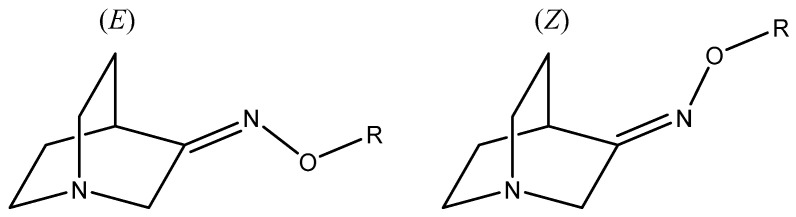
(*E*) and (*Z*) stereoisomers of *O*-substituted quinuclidin-3-one oximes.

Oximes and their derivatives are important intermediates and building blocks for the synthesis of diverse biologically active compounds (e.g., some antimicrobial agents [1], nerve agents [2], antifungal agents [3], insecticides [4], and vasodilators [5]). They are important precursors for various nitrogen-based organic compounds including amines [6], amides [7], nitriles [8], and nitro [9] compounds. In common with many other compounds, stereochemistry plays an important role in the bioactivity of oximes [3] since two possible stereoisomers can be produced in the addition reactions (Figure 1). Therefore, the ability to understand and control the stereochemistry of oxime synthesis is of great importance. The common method for the preparation of oxime derivatives is the reaction of the carbonyl compound with hydroxylamine hydrochloride [10]. This classical method usually requires long reaction times, high temperatures, and the use of organic solvents which can be environmentally harmful. These reactions usually lead to a mixture of (*E*) and (*Z*)-stereoisomers which can have different bioactivity and are usually difficult to separate [11].

**Figure 2:**
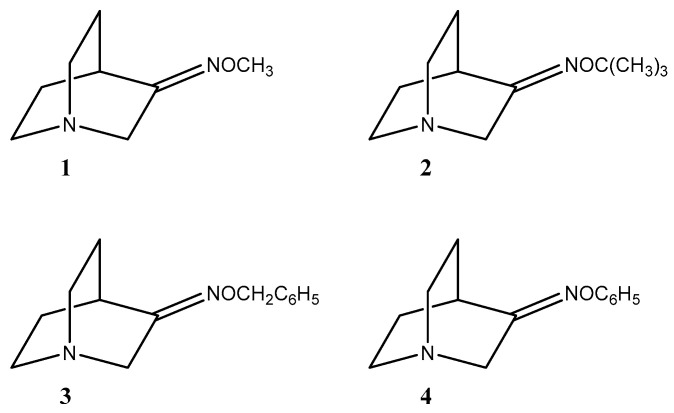
Synthesized *O*-substituted quinuclidin-3-one oximes **1**–**4**.

That is the reason why other methods such as mechanochemistry and microwave-assisted synthesis have been used for oxime synthesis. The aim was to investigate the stereoselectivity of reactions and the influence of the reaction conditions on (*E*) and (*Z*) ratios. For this purpose, three different methods were used and compared: solvent [10], mechanochemical [12,13] and microwave [14,15] synthesis. Recent studies reported mechanochemical and microwave syntheses of oxime heterocyclic compounds and defined the stereoselectivity of those reactions [16,17,18]. Mechanochemical synthesis offers a green alternative to solvent synthesis since it excludes the use of environmentally harmful organic solvents [19,20]. On the other hand, microwave synthesis produces enormous accelerations in the reaction rate; a reaction that takes several hours under conventional conditions can be completed over the course of a few minutes. In this work, four *O*-substituted oximes of quinuclidin-3-one were synthesized [21,22] (Figure 2).

## 2. Results and Discussion

### 2.1. Synthesis of Compounds

It is known that a common method for oxime ether synthesis is using a carbonyl compound with appropriately substituted hydroxylamine hydrochloride in slightly acidic or basic conditions in an aqueous or alcoholic solvent [10,23]. In this research we synthesized methyl (**1**), *tert*-butyl (**2**), benzyl (**3**), and phenyl (**4**) *O*-substituted oximes of quinuclidin-3-one (Figure 2). Although synthesis of compounds **1**, **3** and **4** has already been described in the literature [21], only partial analytical data were presented for novel compounds. Furthermore, the stereoselectivity of the reaction was not considered. No synthetic procedure and/or analytical data were found for compound **2**. Thus, classical solvent syntheses of compounds **1**–**4** in mild conditions at room temperature using quinuclidin-3-one as a starting compound were conducted and a mixture of (*E*)- and (*Z*)-stereoisomers of hydrochloride salts was obtained (Table 1). On the other hand, the use of quinuclidin-3-one hydrochloride gave pure (*E*) stereoisomers for products **3** and **4**, but a significantly prolonged reaction time was needed (Table 1). Therefore, we were interested in finding a new approach that would not result in the formation of the stereoisomer mixture. An earlier study described the mechanochemical synthesis of non-substituted quinuclidin-3-one oxime [12]; we therefore decided to try out the mechanochemical approach as well as microwave-assisted synthesis to synthesize oxime ethers.

**Figure 3:**
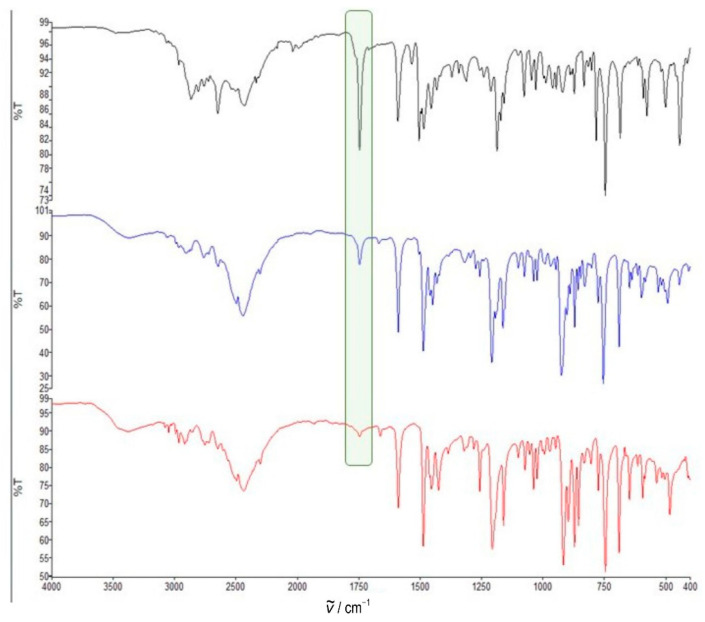
Monitoring of mechanochemical reaction of quinuclidin-3-one hydrochloride and *O*-phenylhydroxylamine hydrochloride with potassium carbonate (product **4·HCl**) by ATR spectroscopy of the crude reaction mixture (black, 1 min, blue, 3 min, red, 6 min). Carbonyl peaks at 1750 cm^−1^ are highlighted with a green rectangle.

**Figure 4:**
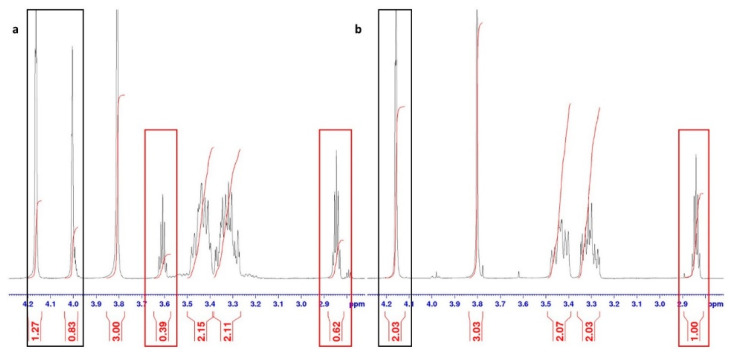
Part of the ^1^H NMR spectrum showing (**a**) mixture of (*E*/*Z*) isomers of compound **1·HCl**, and (**b**) pure (*E*) isomer of product **1·HCl**. Red rectangles indicate the signals for the proton H4, and black rectangles the signals for the H2 protons of the quinuclidine ring.

The ball milling of carbonyl compounds was performed at room temperature with a slight excess (5–10%) of *O*-substituted hydroxylamine hydrochloride. Both dry milling and liquid-assisted grinding (LAG) were performed, since it is known that even a very small amount (a drop) of liquid can significantly accelerate mechanochemical reactions [24]. We applied different solvents (ethanol, hexane and pyridine) and bases (sodium hydroxide and potassium carbonate) in order to monitor the effects of reaction conditions on the isomer ratio. Carbonyl compounds, both quinuclidin-3-one and quinuclidin-3-one hydrochloride were used as the starting material, and products were obtained as hydrochloride salts. It was shown earlier that in the case of quinuclidine carbonyl compounds it is not necessary to use an additional base because the quinuclidine nitrogen atom can serve as one, even when protonated [12]. Reactions were monitored by ATR spectroscopy (Figure 3) by examining the disappearance of the carbonyl peak (around 1750 cm^−1^). The isomer ratio was determined from ^1^H NMR data (Figure 4). The quintet corresponding to the proton H4 was positioned around 2.80 ppm for the (*E*) isomer and around 3.60 ppm for the (*Z*) isomers. The isomer ratio can also be deduced using an H2 singlet that is placed around 4.15 and 4.00 ppm for the (*E*) and (*Z*) isomers, respectively.

Dry grinding of quinuclidin-3-one with *O*-methylhydroxylamine hydrochloride resulted in a 56:44 (*E*/*Z*) mixture of compound **1·HCl** after 60 min (Table 2). However, LAG with ethanol and hexane lowered the reaction time significantly and the reaction was completely stereoselective toward the (*E*) isomer. The reaction of quinuclidin-3-one hydrochloride and *O*-methylhydroxylamine hydrochloride gave no product which can be explained by the lack of base used for the formation of the nucleophile. It was observed that when using the additional base, sodium hydroxide, only one isomer is obtained; however, adding ethanol (LAG) lowered the stereoselectivity.

In the mechanochemical syntheses of oximes **2·HCl**, **3·HCl** and **4·HCl** using quinuclidin-3-one as a starting compound (Table 2), the lowest stereoselectivity was observed. Just as in the case of compound **1·HCl**, using quinuclidin-3-one hydrochloride when an external base was added, reactions without LAG were stereospecific (Table 2).

The final method used for the synthesis of hydrochlorides of compounds **1**–**4** was microwave-assisted synthesis. Microwave-assisted synthesis has a large impact on synthetic organic chemistry. Microwave-enhanced organic synthesis typically reduces reaction times and sometimes improves product yields [14]. In order to determine the influence of reaction conditions on the product isomer ratio, several microwave-assisted reactions were conducted (Table 3). The conversion of reactants in all reactions was complete except for compound **4·HCl**. In general, reactions in polar solvents were shown to be shorter, due to the ability of polar solvents to better absorb microwave energy; thus, polar solvents were used [25]. In the synthesis of compound **1·HCl**, it was shown that stereospecificity was obtained in reactions with a protonated and neutral starting carbonyl compound, with or without the presence of the external base. However, it was also observed that lowering the reaction temperature results in reduced stereoselectivity.

### 2.2. Quantum Chemical Calculations

#### 2.2.1. Reaction Mechanism

**Figure 5:**
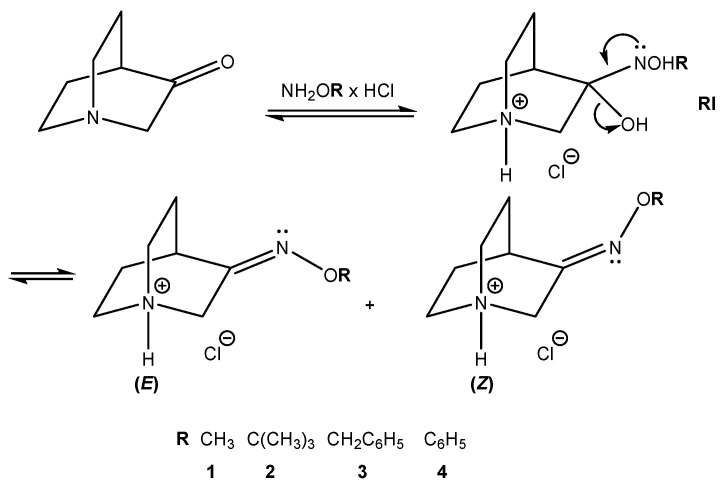
Proposed mechanism of the studied reaction for the synthesis of *O*-substituted quinuclidin-3-one oximes (**1**–**4**).

Mechanisms of the studied reaction for the synthesis of hydrochlorides of *O*-substituted quinuclidin-3-one oximes **1**–**4** are depicted in Figure 5. Upon addition of *O*-substituted hydroxylamine to the carbonyl group, an unstable reaction intermediate (**RI**) is formed. Elimination of the protonated hydroxyl group results in the formation of hydrochloride salts of (*E*)- and (*Z*)-stereoisomers (fast reaction step). In acidic conditions, the protonation of the nitrogen atom follows with the transfer of the proton via an explicit water molecule to the hydroxyl group [26,27].

**Figure 6:**
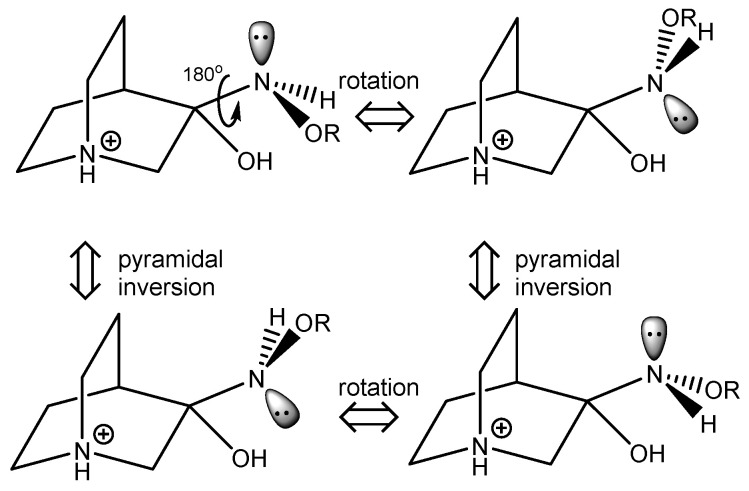
Polytopal rearrangement of **RI** conformers relevant to the formation of products.

Some conformers of the **RI** relevant to the reaction and their stereoisomerization are depicted in Figure 6. Upon rotation of the C–N bond and polytopic rearrangement of the amine group, another conformer essential for the elimination reaction to occur is formed. Since all three ligands connected to the nitrogen atom are different, pyramidal inversion interconverts between enantiomers.

#### 2.2.2. Conformational Analysis

**Figure 7:**
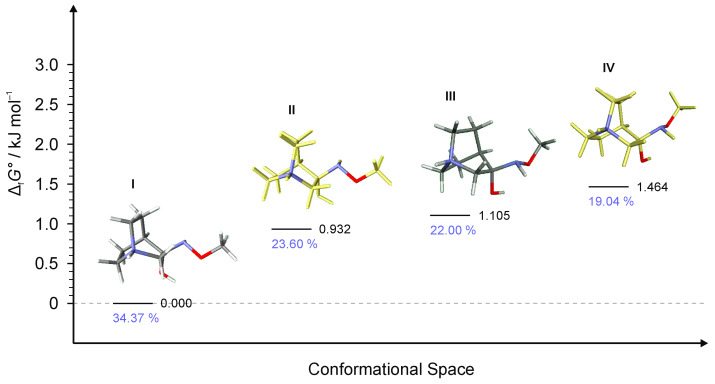
Four lowest energy conformers of **1·HCl** and their relative standards Gibbs energies of formation Δ_f_*G*°calculated at the B3LYP-D3/6-311++G(d,p) level of the theory. (Relative fractions according to Boltzmann distribution at 298.15 K are given in blue.)

**Figure 8:**
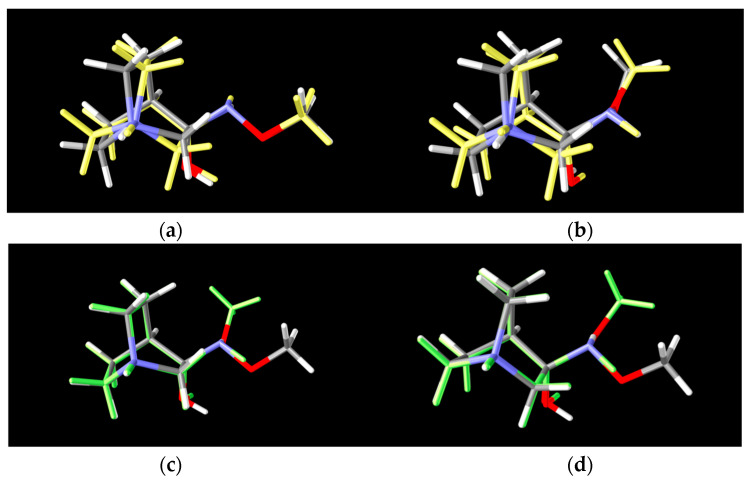
Lowest energy conformers of two polytopes of **RI**-**1**: (**a**) 2 conformers of the 1st-polytope that differ in bicyclic configuration of the quinuclidine ring (**RI1**-**I** and **RI1**-**II**), (**b**) 2 conformers of the 2nd-polytope that differ in bicyclic configuration of the quinuclidine ring (**RI1**-**III** and **RI1**-**IV**) (grey: lower energy conformer, yellow: higher energy conformer), (**c**) 2 lower energy polytopes that differ in spatial arrangements of the amino group (**RI1**-**I** and **RI1**-**III**) and (**d**) 2 higher energy polytopes that differ in spatial arrangements of the amino group (**RI1**-**II** and **RI1**-**IV**) (grey: lower energy polytope, green: higher energy polytope).

**Figure 9:**
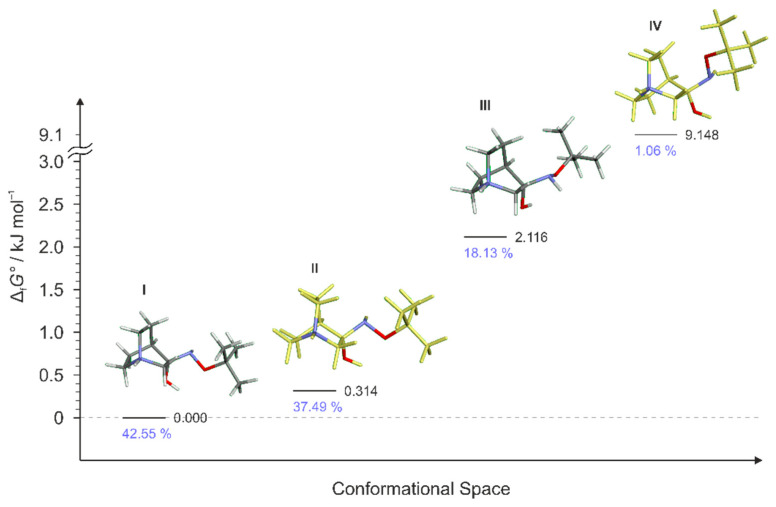
Four lowest energy conformers of **RI2** and their relative standards Gibbs energies of formation Δ_f_*G*°calculated at the B3LYP-D3/6-311++G(d,p) level of the theory. (Relative fractions according to Boltzmann distribution at 298.15 K are given in blue.)

**Figure 10:**
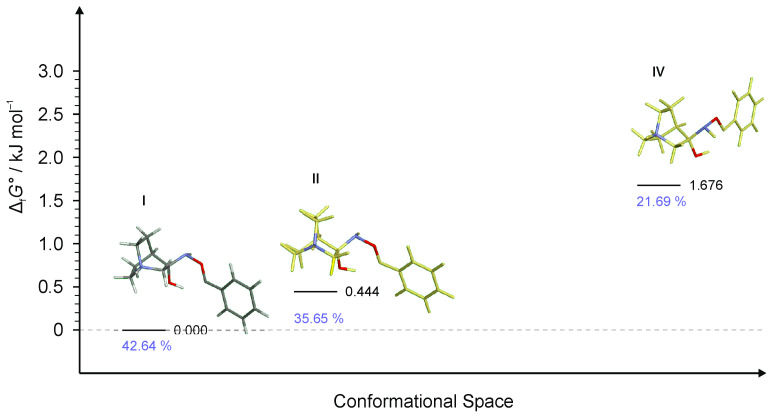
Lowest energy conformers of **RI3** and their relative standards Gibbs energies of formation Δ_f_*G*°calculated at the B3LYP-D3/6-311++G(d,p) level of the theory. (Relative fractions according to Boltzmann distribution at 298.15 K are given in blue. Conformer **RI3**-**III** was not found.)

**Figure 11:**
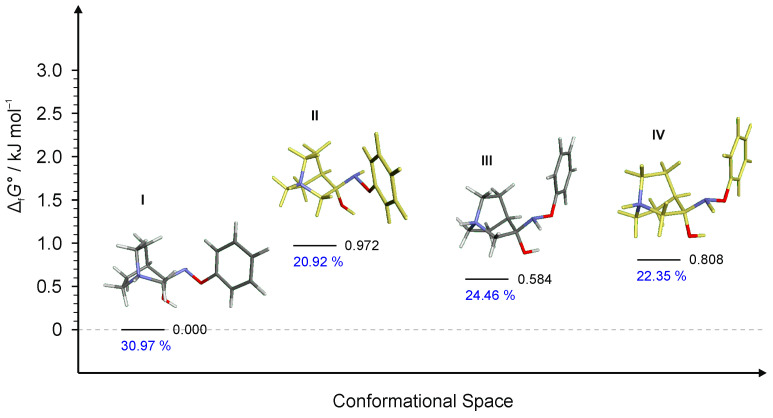
Four lowest energy conformers of **RI4** and their relative standards Gibbs energies of formation Δ_f_*G*°calculated at the B3LYP-D3/6-311++G(d,p) level of the theory. (Relative fractions according to Boltzmann distribution at 298.15 K are given in blue.)

To explain the existence of two obtained stereoisomers in chemical syntheses, full conformational analyses of the reaction intermediates **RI1**–**RI4** (Figure 5) were performed. Full conformational spaces for all intermediates were determined by the systematic scan of the potential energy surface spanned in all possible torsional degrees of freedom (Figure 7, Figure 8, Figure 9, Figure 10 and Figure 11). The goal of each conformational analysis was to confirm that those conformers can lead to synthesized products in chemical reactions. Then, using the different standard Gibbs energies of formation for the intermediate conformers, it was possible to estimate the stereoselectivity of products. These findings were compared with experimental data and those calculated from standard Gibbs energies of formation for stereoisomers of hydrochlorides **1**–**4** (Table 4).

For **RI1**, a total of 12 conformers were found but only four of them (Figure 7) were energetically relevant (others were too high in energy and their relative content was close to zero). These four conformers can be divided into two groups of polytopes with the only difference between them being the configuration of the amino group. In addition, these two groups of polytopes were found for all reaction intermediates **RI2**–**RI4** (Figure 9, Figure 10 and Figure 11). The lowest energy polytope configuration in all cases lead to the (*E*)-products (conformers **RI1**-**I** and **RI1**-**II**), whereas the second one leads to the (*Z*)-products (conformers **RI1**-**III** and **RI1**-**IV)** (Figure 7). This belongs to a classic case of the Curtin–Hammett principle [28].

Conformers **RI1**-**I** and **RI1**-**II** belong to the polytope group that will lead into the (*E*)-**1·HCl** product, and they differ in the conformation of the bicyclic ring (Figure 8a). Conformers **RI1**-**III** and **RI1**-**IV** belong to the other polytope that leads into the (*Z*)-**1·HCl** product and, again, the difference between them is only in the conformation of the bicyclic ring (Figure 8b).

Multiple conformers were found for **RI2** and **RI3** intermediates but in both cases, only three of them were energetically relevant (Figure 9 and Figure 10). Conformer **RI2**-**IV** was also very high in energy and its relative content was only 1% whereas for **RI3** only three conformers were found (conformer **RI3**-**III** was not found even when using a very small optimization step or higher basis set). In each case, the first conformer of the lowest energy led to the (*E*)-product.

For **RI4** a total of 18 conformers were found but only four of them were energetically relevant (others were very high in energy and their relative content was close to zero). As in the previous cases, these four conformers can be divided into two groups of polytopes with the only difference between them being the configuration of the amino group. The first polytope configuration leads to the (*E*)-**4** product (conformers **RI4**-**I** and **RI4**-**II**), whereas the second one leads to the (*Z*)-**4** product (conformers **RI4**-**III** and **RI4**-**IV**) (Figure 11). Conformers **RI4**-**I** and **RI4**-**II** belong to the polytope group that leads into the (*E*)-**4** product and they differ in the conformation of the bicyclic ring (Figure 8a). Conformers **RI4**-**III** and **RI4**-**IV** belong to the other polytope that leads into the (*Z*)-**4** product and again the difference between them is only in the conformation of the bicyclic ring (Figure 8b). Structures of all conformers are available in Supplementary Materials.

#### 2.2.3. Reaction Products and Stereoselectivity

Quantum chemical calculations of the reaction products and some transition states were performed at the B3LYP-D3/6-311++G(d,p) level of the theory. Calculations were conducted in acidic conditions for protonated products. In each case of the compounds **1**–**4,** the (*Z*)-products were much higher in energy than the corresponding (*E*)-products (Table 4). The stereoselectivity was shifted in the direction of thermodynamically more stable (*E*)-products. Conducting reactions using mechanochemical and/or microwave synthesis resulted in reaction acceleration and thermodynamic reaction control. In these cases, the thermodynamically favoured product was obtained in large excess.

In classical syntheses that lasted only a few minutes, reactions were kinetically controlled by the corresponding transition state energies. Standard Gibbs energies of activation were calculated for transition states of **1** in complex with one explicit water molecule. These values were 20.29 and 5.30 kJ mol**^–1^** for polytopes **RI1**-**I** and **RI1-III**, respectively. The least stable polytope, **RI1-III**, reacts faster than the energetically preferred polytope **RI1-I**. Obtained product ratios were determined by the activations energies and similar values can be expected for other compounds.

## 3. Materials and Methods

Reagents and solvents used for the synthesis were purchased from Sigma-Aldrich, Co. (St. Louis, MO, USA), and used without further purification. Quinuclidin-3-one was prepared from quinuclidin-3-one hydrochloride with sodium hydroxide in water. Mechanochemical syntheses of the compounds were performed on an IST 500 ball mill (InSolido Technologies, Zagreb, Croatia) in Teflon jars (volume of 7 mL) with two zirconium balls (*r* = 3.5 mm) and a grinding frequency of 25 Hz. Microwave synthesis was carried out in the CEM Focused Microwave TM Synthesis System, Discover SP (Matthews, NC, USA). The progress of reactions was monitored by IR spectroscopy (Perkin Elmer FT-IR Spectrum Two with Diamond ATR Attachment, Waltham, MA, USA) and visualized in Spectrum Software, v. 10.3.4. (Perkin Elmer, Waltham, MA, USA). ^1^H NMR and ^13^C NMR spectra were recorded using a Bruker Avance III HD Ascend NMR spectrometer (Billerica, MA, USA) at 400 MHz (^1^H NMR) and 100 MHz (^13^C NMR) in deuterated D_2_O at room temperature and were the same for neutral compounds and their hydrochloride salts. All NMR spectra were visualized in Brucker TopSpin 3.6.2. Chemical shifts (*δ*) were expressed in parts per million (ppm) relative to the internal standard tetramethylsilane (TMS). Quinuclidin-3-one protons and carbons were named according to standard quinuclidin-3-one numeration (Ha for axial protons, Hb for equatorial protons) while benzyl protons and carbons have the abbreviation *bzl*. The melting points of the obtained hydrochloride products were determined with a Büchi Melting Point B-545. High-resolution mass spectra (HRMS) analysis was performed on NanoUHPLC-MS/MS ThermoScientific QEcaxtive Plus (Waltham, MA, USA).

### 3.1. Synthesis of Compounds

Four hydrochloride salts of substituted oximes were synthesized using quinuclidin-3-one and quinuclidin-3-one hydrochloride. Three methods were used for the oxime preparation: Method A—classical synthesis in solution, Method B—mechanochemical synthesis and Method C—microwave synthesis. For the synthesis of neutral compounds, solvent if present was evaporated, water saturated with potassium carbonate was added, followed by chloroform extraction. Hydrochloride salts were obtained by recrystallization from ethanol.

Method A—Classical synthesis in solution

Quinuclidin-3-one or quinuclidin-3-one hydrochloride (1.6 mmol mg, 1 eq) was dissolved in ethanol and appropriate *O*-substituted hydroxylamine hydrochloride (1.05 eq) was added. The reaction was stirred at room temperature. Detailed data are given in Table 1.

Method B—Mechanochemical synthesis

A mixture of quinuclidin-3-one or quinuclidin-3-one hydrochloride (0.16 mmol, 1 eq) and corresponding *O*-substituted hydroxylamine hydrochlorides (1.05 eq) was milled for 3–60 min. Liquid-assisted grinding (LAG) was also carried out, using 30 µL of ethanol, hexane, pyridine or water, respectively. Bases such as sodium hydroxide and potassium carbonate were also used in several experiments. Detailed data are given in Table 2.

Method C—Microwave synthesis

A mixture of quinuclidin-3-one or quinuclidin-3-one hydrochloride (0.5 mmol, 1 eq) and corresponding *O*-substituted hydroxylamine hydrochlorides (1.05 eq) and solvent (0.5 mL of acetone, acetonitrile, pyridine, or absolute ethanol, respectively) was stirred under microwave irradiation (250 W) for 1–3 min. Detailed data are given in Table 3.

#### 3.1.1. Quinuclidin-3-one *O*-Methyloxime (**1**)

Colourless oil, IR *ῦ*/cm^−1^: 2945, 2870 (C-H), 1455 (C = N), 1040 (N-O); **1**·HCl, white solid, melting point 224.0–226.5 °C. IR *ῦ*/cm^−1^: 2970 (C-H), 2175–2620 (+N-H), 1440 (C = N), 1030 (N-O). ^1^H NMR (400 MHz, D_2_O) *δ*/ppm: 1.96–2.05 (m, 2H, H5a, H7a), 2.09–2.20 (m, 2H, H5b, H7b), 2.84 (quintet, *J^1,2^* = 3.2 Hz, 1H, H4), 3.26–3.35 (m, 2H, H6a, H8a), 3.39–3.45 (m, 2H, H6b, H8b), 3.80 (s, 3H, CH_3_), 4.15 (s, 2H, H2a, H2b). ^13^C NMR (100 MHz, D_2_O) *δ*/ppm: 21. 67 (C5, C7), 26.57 (C4), 46.99 (C6, C8), 50.63 (C2), 61.73 (CH_3_). HRMS (*m*/*z*): calculated 155.1184 (C_8_H_15_N_2_O), found 155.1181. Figures S1–S3, S13 and S17.

#### 3.1.2. Quinuclidin-3-one *O*-Tert-butyloxime (**2**)

Colourless oil, IR *ῦ*/cm^−1^: 2945, 2870 (C-H), 1455 (C = N), 1030 (N-O); **2**·HCl, white solid, melting point 238.9–241.1 °C. IR *ῦ*/cm^−1^: 2970 (C-H), 2135–2625 (+N-H), 1450 (C = N), 1035 (N-O). ^1^H NMR (400 MHz, D_2_O) δ/ppm: 1.23 (s, 9H, tBu), 1.96–2.07 (m, 2H, H5a, H7a), 2.11–2.21 (m, 2H, H5b, H7b), 2.90 (quintet, *J^1,2^* = 3.2 Hz, 1H, H4), 3.26–3.36 (m, 2H, H6a, H8a), 3.39–3.50 (m, 2H, H6b, H8b), 4.16 (s, 2H, H2a, H2b). ^13^C NMR (100 MHz, D_2_O) δ/ppm: 21. 77 (C5, C7), 26.54 (C(**C**H_3_)_3_) 26.65 (C4), 46.97 (C6, C8), 51.02 (C2), 80.19 (**C**(CH_3_)_3_), 154.56 (C3). HRMS (*m*/*z*): calculated 197.1654 (C_11_H_21_N_2_O), found 197.1652. Figures S4–S6, S14 and S18.

#### 3.1.3. Quinuclidin-3-one *O*-Benzyloxime (**3**)

Colourless oil, IR *ῦ*/cm^−1^: 2945, 2865 (C-H), 1455 (C = N), 1030 (N-O); **3**·HCl, white solid, melting point 194.4–197.5 °C. IR *ῦ*/cm^−1^: 2970 (C-H), 2150- 2600 (+N-H), 1450 (C = N), 1030 (N-O); ^1^H NMR (400 MHz, D_2_O) δ/ppm: 1.90–2.00 (m, 2H, H5a, H7a), 2.07–2.18 (m, 2H, H5b, H7b), 2.81 (quintet, *J^1,2^* = 3.2 Hz, 1H, H4), 3.22–3.32 (m, 2H, H6a, H8a), 3.37–3.47 (m, 2H, H6b, H8b), 4.18 (s, 2H, H2a, H2b), 5.06 (s, 2H, CH_2_ bzl), 7.31–7.44 (m, 5H, H2, H3, H4, H5, H6 bzl). ^13^C NMR (100 MHz, D_2_O) δ/ppm: 21. 61 (C5, C7), 26.61 (C4), 46.95 (C6, C8), 50.82 (C2), 51.87 (CH_2_ bzl), 128.30 (C2, C6 bzl), 185.52 (C4 bzl), 128.78 (C3, C5 bzl), 136.93 (C1 bzl), 155.67 (C3). HRMS (m/z): calculated 231.1497 (C_14_H_19_N_2_O), found 231.1495. Figures S7–S9, S15 and S19.

#### 3.1.4. Quinuclidin-3-one *O*-Phenyloxime (**4**)

Brown oil, IR *ῦ*/cm^−1^: 2970 (C-H), 1455 (C = N), 1030 (N-O); **4**·HCl, brown solid, melting point 153.1–156.5 °C, IR *ῦ*/cm^−1^: 3050 (C-H), 1480 (C = N), 1075 (N-O). ^1^H NMR (400 MHz, D_2_O) δ/ppm: 2.03–2.14 (m, 2H, H5a, H7a), 2.17–2.28 (m, 2H, H5b, H7b), 3.00 (quintet, *J^1,2^* = 3.2 Hz, 1H, H4), 3.31–3.43 (m, 2H, H6a, H8a), 3.44–3.56 (m, 2H, H6b, H8b), 4.40 (s, 2H, H2a, H2b), 7.08–7.20 (m, 2H, H2, H4, H6 bzl), 7.33–7.40 (m, 2H, H3, H5 bzl). ^13^C NMR (100 MHz, D_2_O) δ/ppm: 21. 57 (C5, C7), 26.76 (C4), 47.02 (C6, C8), 50.89 (C2), 115.35 (C2, C6 bzl), 123.57 (C4 bzl), 129.75 (C3, C5 bzl), 158.28 (C3). HRMS (m/z): calculated 217.1341 (C_13_H_17_N_2_O), found 217.1336. Figures S10–S12, S16 and S20.

### 3.2. Quantum Chemical Calculation

A conformational space search for reaction intermediates was performed by analyzing the complete potential energy surfaces (PES) of compounds **RI1**–**RI4**. PES for each synthesized compound was spanned in 4D-space defined by torsional coordinates around single bonds (Figure 6). For **RI1** and **RI2**, the torsional coordinates *φ*_1_, *φ*_2_ and *φ*_3_ were investigated in the relative range 0–360°, whereas the torsional coordinate *φ*_4_ was explored in the relative range 0–120°, providing a total number of 559 872 single-point calculations. For **RI3**, the torsional coordinates *φ*_1_, *φ*_2_, *φ*_3_ and *φ*_4_ were investigated in the relative range 0–360°, whereas *φ*_5_ was explored in the relative range 0–180°, giving a total number of 30 233 088 single point calculations. For **RI4**, the torsional coordinates *φ*_1_, *φ*_2_ and *φ*_3_ were investigated in the relative range 0–360°, whereas *φ*_4_ was explored in the relative range 0–180°, giving a total number of 839 808 single-point calculations. All torsional angles were measured relatively starting from the initial geometry structures. Scans were calculated by varying the torsional coordinates using an automatic conformation generator implemented in *qcc* [29,30]. All single-point calculations were performed using the PM7 Hamiltonian [31].

Energy data from PES scans were arranged in four- or five-way array (four-dimensional or five-dimensional) and parallelized optimization algorithm for the arbitrary number of ways implemented in program *moonee* [32,33] was used to determine all local minima on the investigated PES. All determined local minima geometries were reoptimized at the B3LYP/6-311++G(d,p) level of the theory. To ensure that the obtained geometries indeed corresponded to local minima, harmonic frequency calculations were performed. (*E*)- and (*Z*)-isomers of **1·HCl4·HCl** were created from the corresponding lowest-energy conformers with the elimination of water molecules. Each structure was optimized at the B3LYP/6-311++G(d,p) level of the theory. Harmonic vibrational frequencies were computed to ensure that the structures were local minima on the PES. All quantum-chemical calculations were performed using the Gaussian 16 program [34].

## 4. Conclusions

Synthesis of four novel *O*-substituted quinuclidin-3-one oximes was carried out. Classical, mechanochemical and microwave synthesis was carried out and compared. Mechanochemistry proved to be an excellent alternative for the transformations of quinuclidin-3-one into *O*-substituted oximes using *O*-substituted hydroxylamine hydrochlorides. Conversion to *O*-substituted oxime hydrochloride salt was full, and no solvent was necessary, or only a catalytic amount needed to be added. That makes this route environmentally friendly and a more energy-efficient alternative to the other methods. Another excellent alternative to the solvent synthesis of oximes is microwave-assisted synthesis. The preparation of **1**–**3** hydrochloride salts under microwave irradiation proved to be a fast and facile method with low energy consumption. Both methods provided several reactions with complete stereoselectivity. The use of quinuclidin-3-one hydrochloride and sodium hydroxide provided stereospecific synthesis for all four oximes. Full conformational analyses were performed for the reaction intermediates and the lowest-energy conformers were determined. These conformers that differ in polytope rearrangement on the nitrogen atom of the amino group led to the different reaction products. The benefit of mechanochemical or microwave synthesis lies in the acceleration in the reactions, which facilitates thermodynamic control of the reaction and produces only one product (actually resulting in stereocontrol). Standard Gibbs energies of products confirmed the obtained stereoselectivity in chemical syntheses.

## Figures and Tables

**Table 1:** Classical synthesis in solution (Method A) of compounds **1**–**4**. *E*/*Z* ratio was determined by ^1^H NMR spectroscopy.

Starting Compound	Reaction Time	Product	*E*/*Z*
Quinuclidin-3-one	3 min	**1·HCl**	60:40
	8 min	**2·HCl**	93:7
	5 min	**3·HCl**	70:30
	6 min	**4·HCl**	65:35
Quinuclidin-3-one hydrochloride	24 h	**1·HCl**	85:15
	48 h	**2·HCl**	*
	24 h	**3·HCl**	100:0
	48 h	**4·HCl**	100:0

* No product was detected.

**Table 2:** Reaction conditions for mechanochemical synthesis (Method B) of compounds **1·HCl**–**4·HCl**. *E*/*Z* ratio was determined by ^1^H NMR spectroscopy.

Starting Compound	LAG	Base	Reaction Time/Min	Product	*E*/*Z*
Quinuclidin-3-one	-	-	60	**1·HCl**	56:44
	EtOH	-	5	**1·HCl**	100:0
	Hexane	-	4	**1·HCl**	100:0
	Pyridine	-	30	**1·HCl**	50:50
Quinuclidin-3-one hydrochloride	-	-	*	**1·HCl**	-
	-	NaOH	10	**1·HCl**	100:0
	EtOH	NaOH	10	**1·HCl**	61:39
	-	K_2_CO_3_	5	**1·HCl**	100
Quinuclidin-3-one	-	-	8	**2·HCl**	80:20
	EtOH	-	3	**2·HCl**	75:25
	Hexane	-	3	**2·HCl**	78:22
	Pyridine	-	5	**2·HCl**	75:25
Quinuclidin-3-one hydrochloride	Pyridine	-	3	**2·HCl**	75:25
	-	NaOH	5	**2·HCl**	100:0
	EtOH	NaOH	10	**2·HCl**	70:30
	-	K_2_CO_3_	5	**2·HCl**	100:0
Quinuclidin-3-one	-	-	5	**3·HCl**	67:33
	EtOH	-	5	**3·HCl**	90:10
	Hexane	-	8	**3·HCl**	75:25
	Pyridine	-	8	**3·HCl**	80:20
Quinuclidin-3-one hydrochloride	Pyridine	-	4	**3·HCl**	100:0
	-	NaOH	6	**3·HCl**	100:0
	EtOH	NaOH	10	**3·HCl**	96:4
	-	K_2_CO_3_	4	**3·HCl**	70:30
Quinuclidin-3-one	-	-	6	**4·HCl**	49:51
	EtOH	-	3	**4·HCl**	50:50
	Hexane	-	3	**4·HCl**	60:40
Quinuclidin-3-one hydrochloride	-	-	*	**4·HCl**	-
	-	NaOH	2	**4·HCl**	100:0
	EtOH	NaOH	10	**4·HCl**	72:28
	-	K_2_CO_3_	6	**4·HCl**	100:0

* No product was detected.

**Table 3:** Reaction conditions for microwave synthesis (Method C) of compounds **1·HCl**–**4·HCl**. *E*/*Z* ratio was determined by ^1^H NMR spectroscopy.

Starting Compound	Solvent	Temperature/°C	Base	Time/Min	Product	*E*/*Z*
Quinuclidin-3-one	Acetonitrile	140	-	3	**1** **·HCl**	100
	Acetonitrile	140	-	3	**2** **·HCl**	100
	Acetonitrile	140	-	3	**3** **·HCl**	100
	Acetonitrile	140	-	3 *	**4** **·HCl**	-
	Acetonitrile	140	-	1	**1** **·HCl**	100
	Acetonitrile	80	-	1	**1** **·HCl**	60:40
	Acetone	140	-	5	**1** **·HCl**	100
	Acetone	140	-	1 *	**1** **·HCl**	-
	Ethanol	140	-	3	**2** **·HCl**	90:10
	Ethanol	140	-	3	**1** **·HCl**	65:35
	Ethanol	80	-	1	**1** **·HCl**	60:40
	Ethanol	140	-	3	**3** **·HCl**	85:15
Quinuclidin-3-one hydrochloride	Acetonitrile	140	K_2_CO_3_	3	**1** **·HCl**	100
	Acetonitrile	140	-	3	**1** **·HCl**	100
	Acetonitrile	140	NaOH	3	**1** **·HCl**	100
	Pyridine	140	Pyridine	3	**1** **·HCl**	85:15
	Ethanol	140	NaOH	3	**1** **·HCl**	55:45

* No product was detected.

**Table 4:** Calculated differences of standard Gibbs energies of formation ΔΔ_f_Gº relative to the (*E*)-isomers for hydrochlorides **1**–**4** (B3LYP-D3/6-311++G(d,p)).

Compound	ΔΔ_f_*G*°/kJ mol^–1^
**(*E*)-1** **·HCl**	0.00
**(*Z*)-1** **·HCl**	13.77
**(*E*)-2** **·HCl**	0.00
**(*Z*)-2** **·HCl**	10.34
**(*E*)-3** **·HCl**	0.00
**(*Z*)-3** **·HCl**	8.84
**(*E*)-4** **·HCl**	0.00
**(*Z*)-4** **·HCl**	10.41

## Data Availability

Not applicable.

## Supplementary Materials

The following supporting information can be downloaded at: https://www.mdpi.com/article/10.3390/ijms232012331/s1.
