# Supplementary material for: Polytopal Rearrangement Governing Stereochemistry of Bicyclic Oxime Ether Synthesis
Source: Int J Mol Sci. 2022 Oct 15;23(20):12331. doi: 10.3390/ijms232012331 (PMC9604001; doi:10.3390/ijms232012331)
Supplement: Supplementary file 1 [file ijms-23-12331-s001.zip › ijms-1910476-supplementary.pdf]

Supplementary Materials

Contents

Supplementary Figures

Figures S1-S12: NMR spectra (^{1}H, ^{13}C) 1

Figures S13-S16: IR spectra 13

Figures S17-S20: HRMS spectra 17

Cartesian coordinates of conformers 19

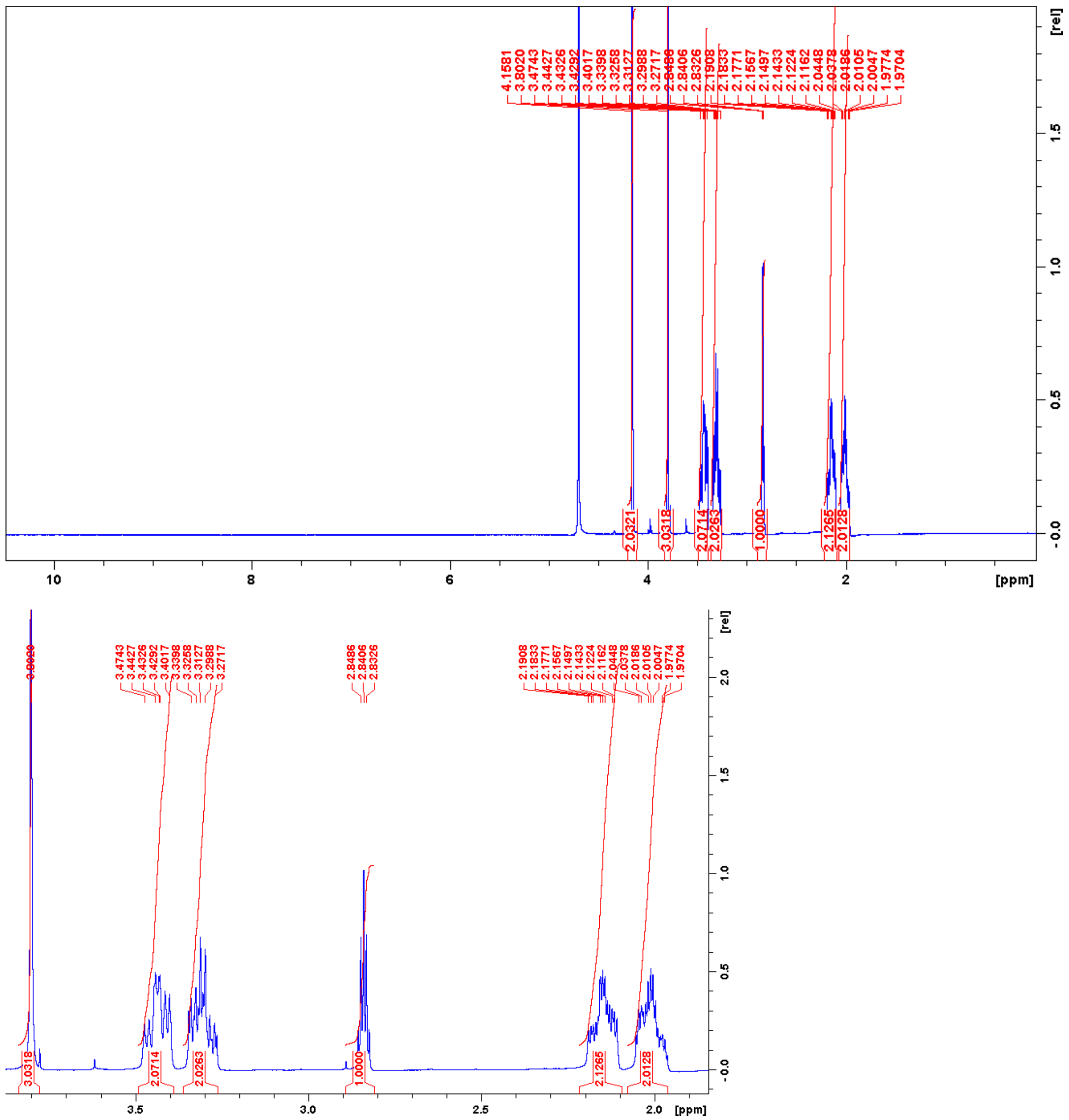

Figure S1. ^{1}H NMR spectrum of (*E*)-**1**.

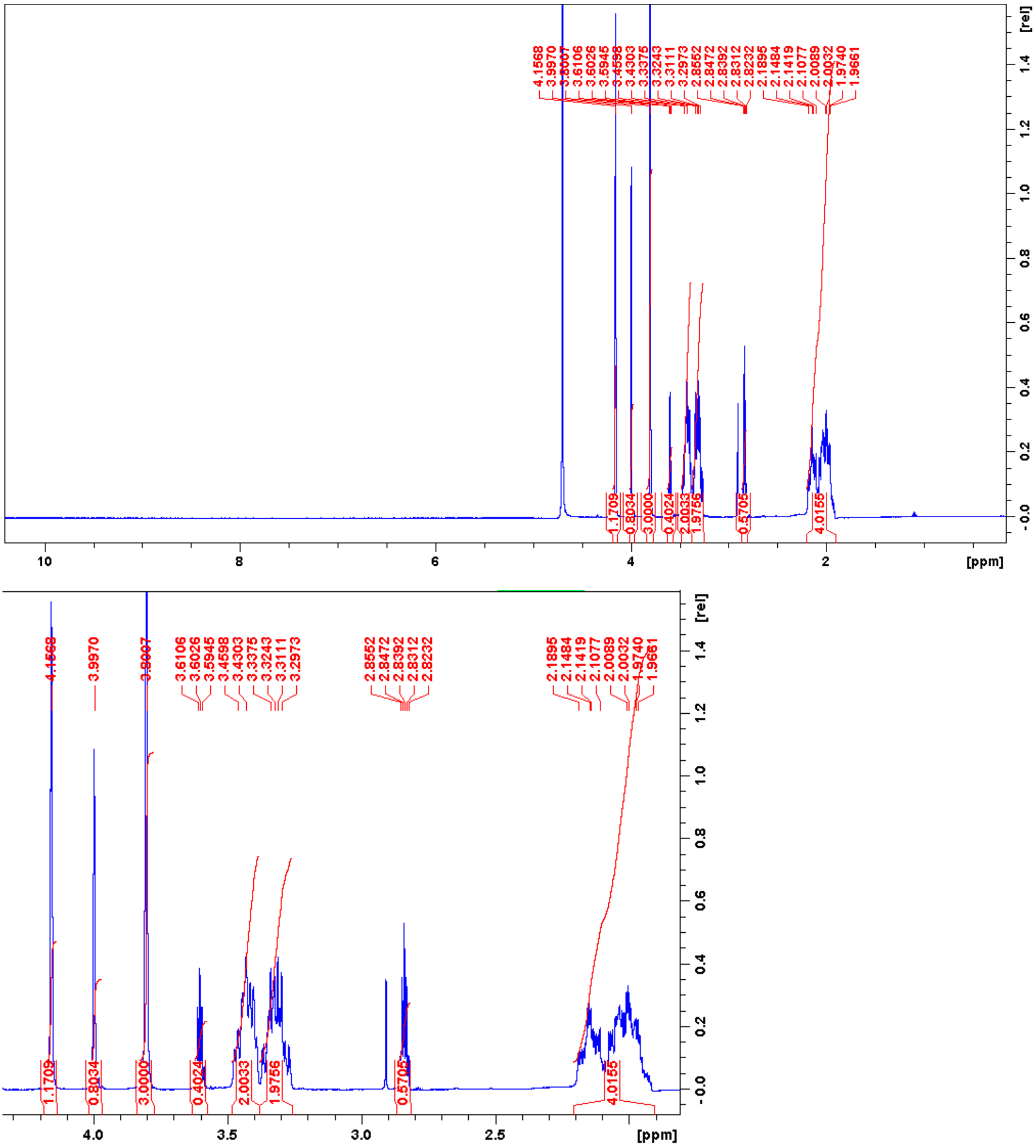

Figure S2. ^{1}H NMR spectrum of (*E*)- and (*Z*)-**1**.

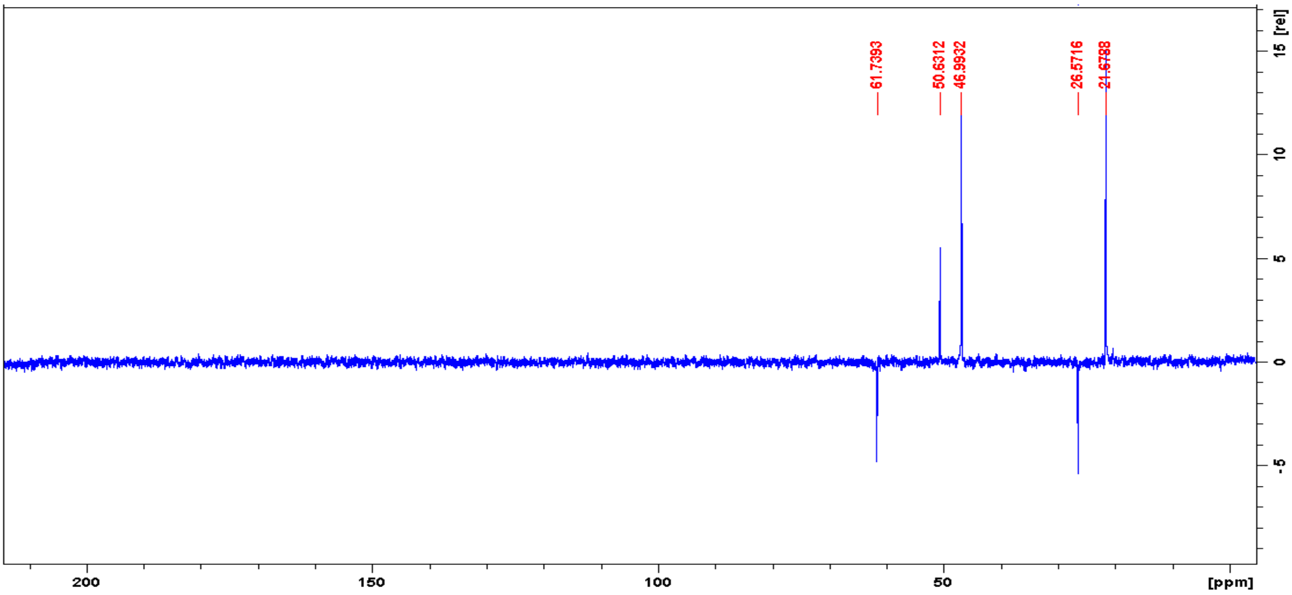

Figure S3. ^{13}C NMR spectrum of (*E*)-**1**.

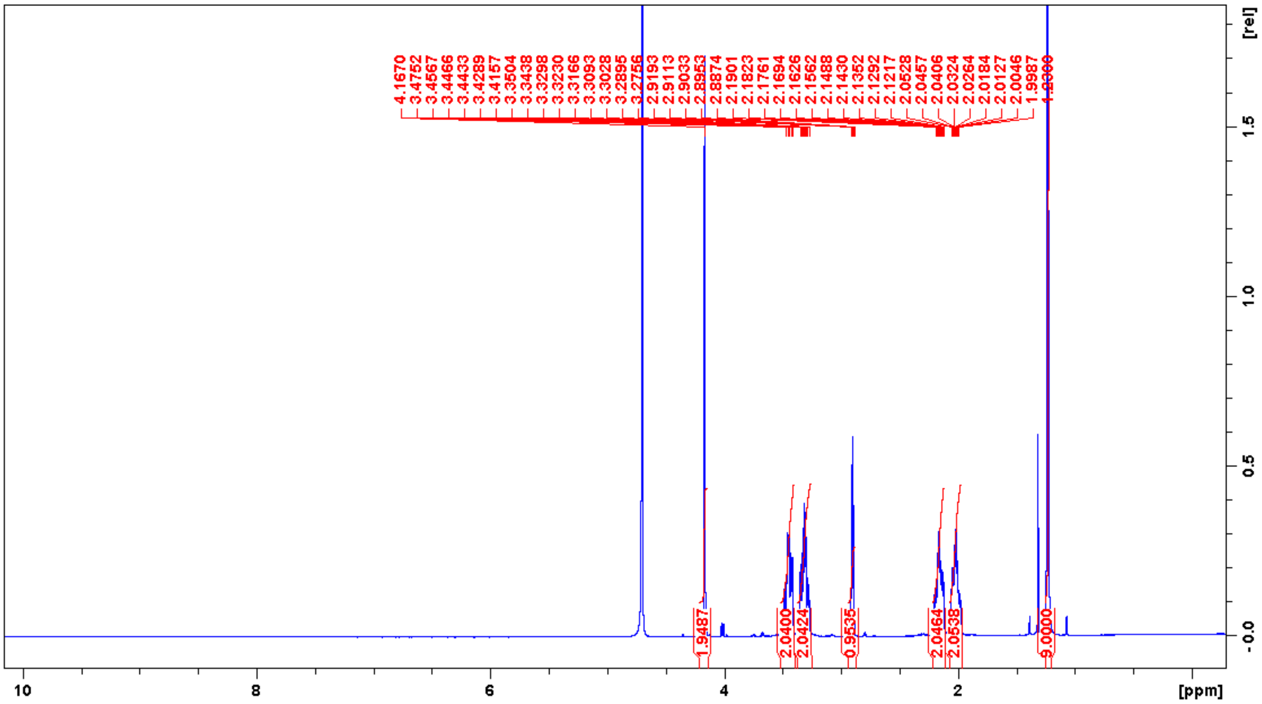

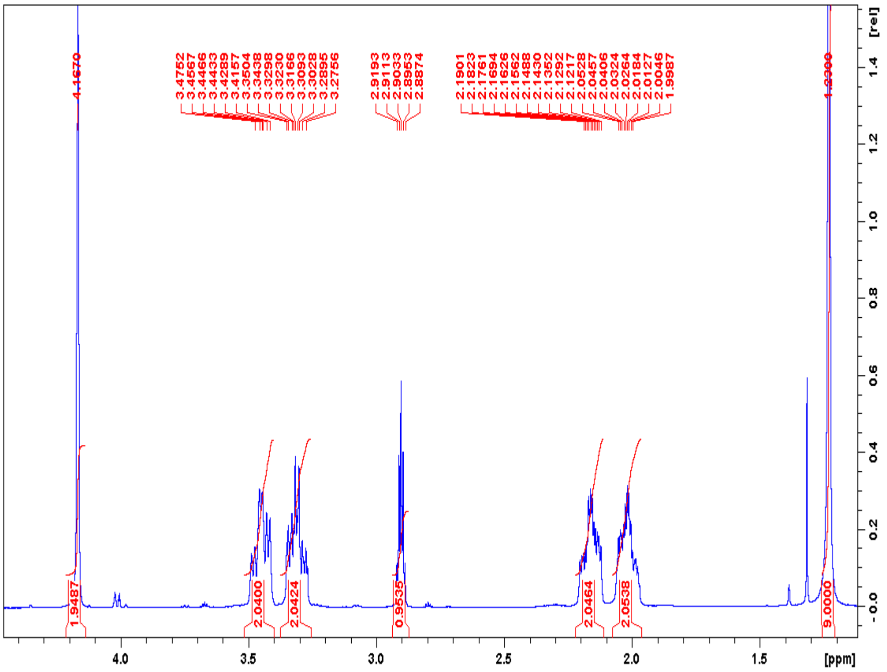

Figure S4. ^{1}H NMR spectrum of (*E*)-**2**.

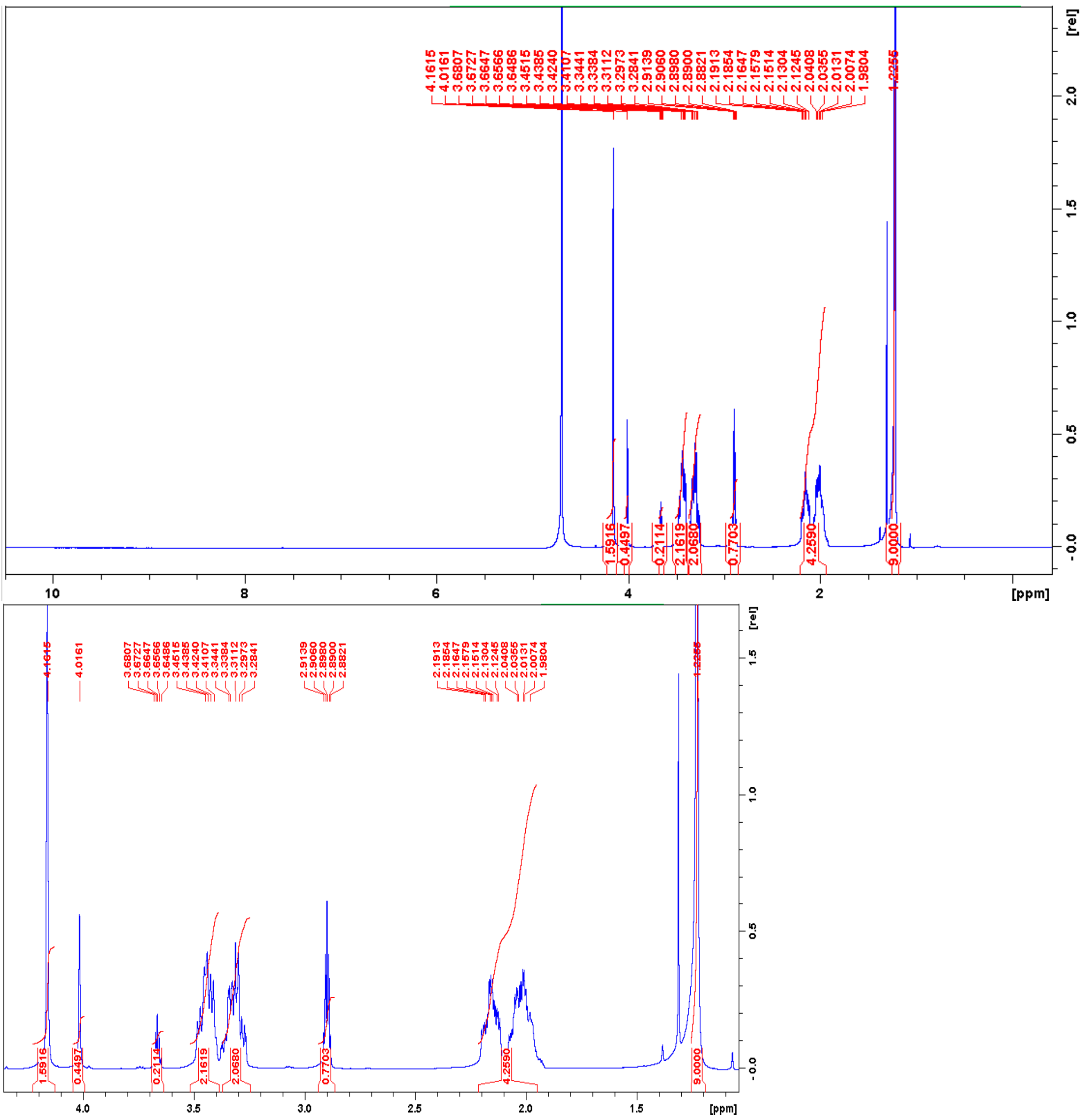

Figure S5. ^{1}H NMR spectrum of (*E*)- and (*Z*)-**2**.

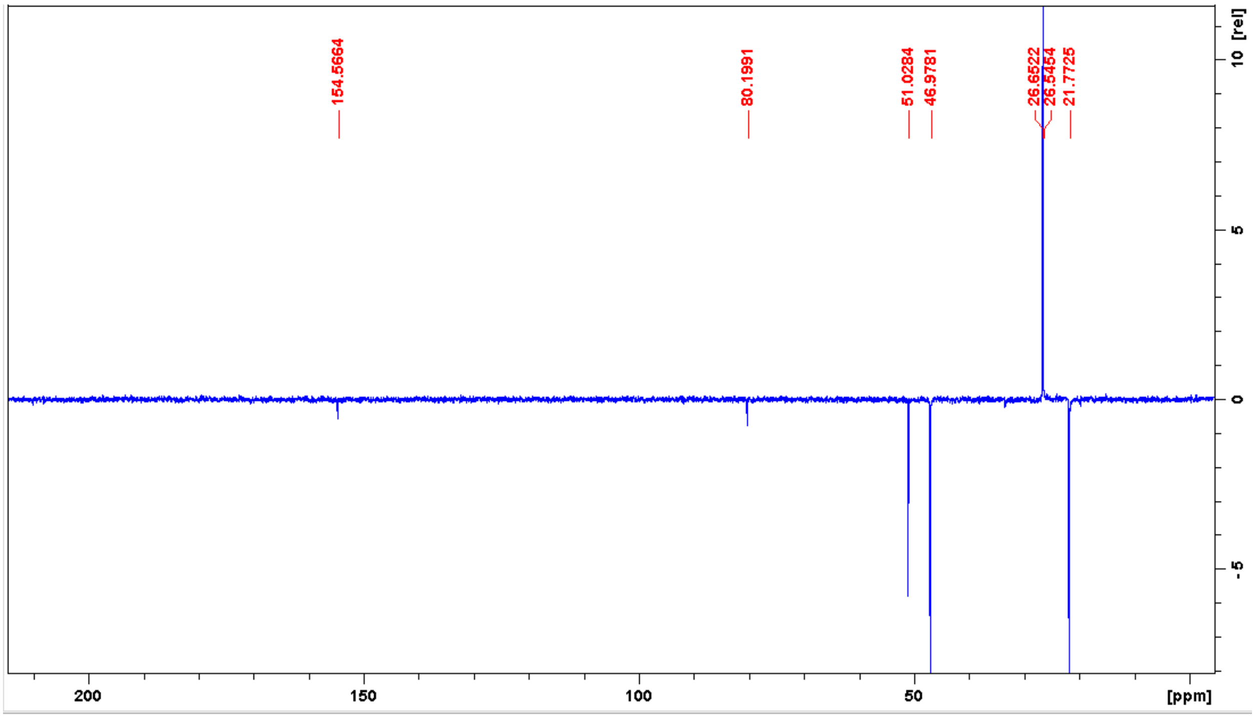

Figure S6. ^{13}C NMR spectrum of (*E*)-**2**.

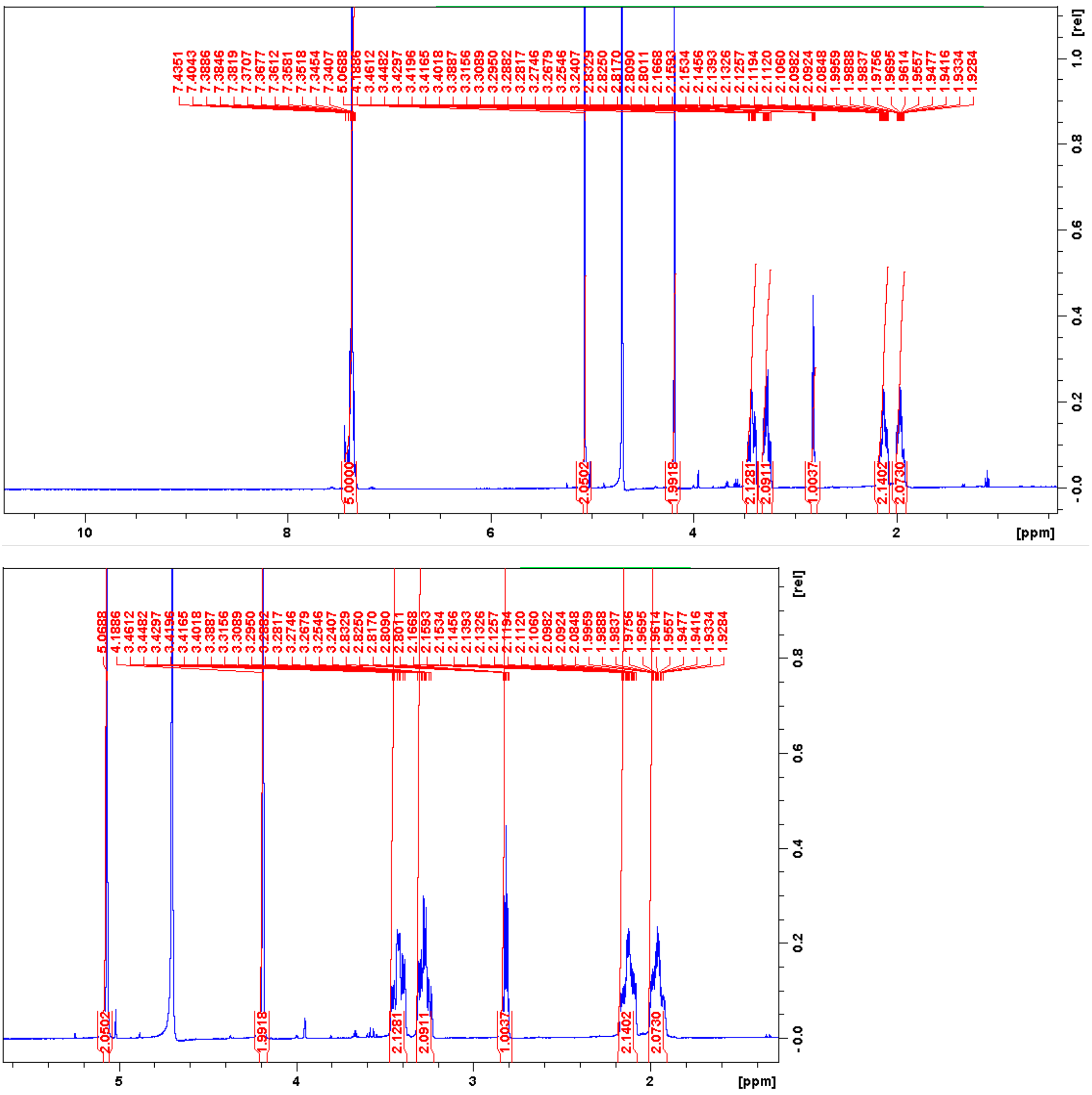

Figure S7. ^{1}H NMR spectrum of (*E*)-**3**.

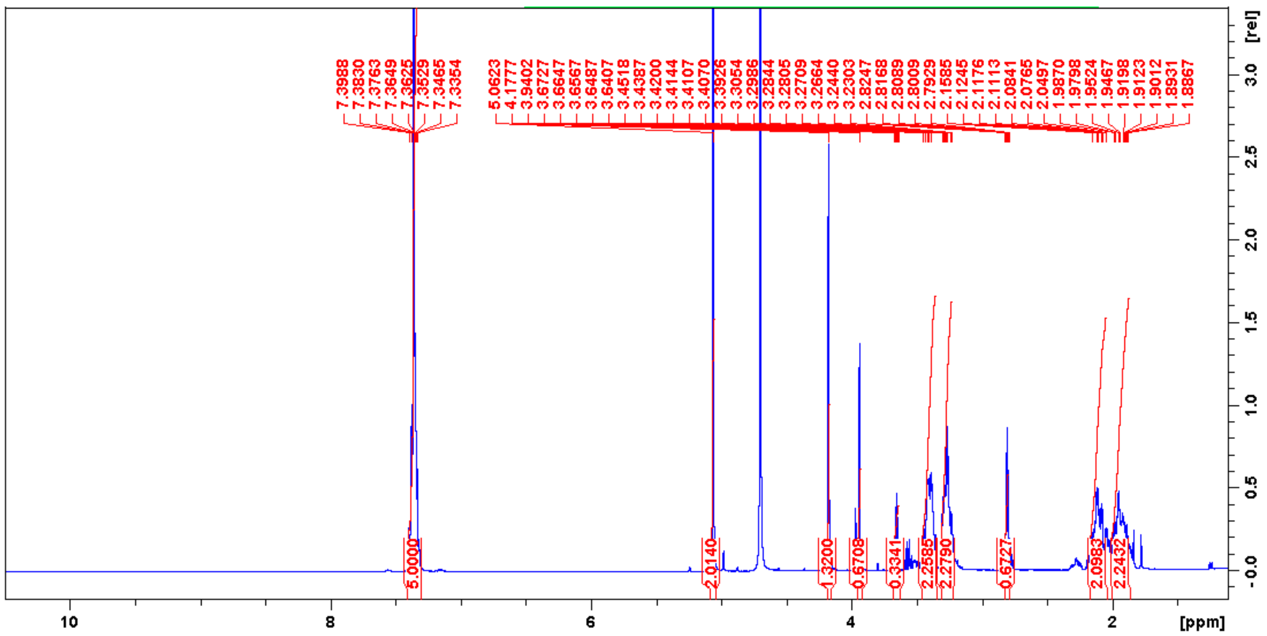

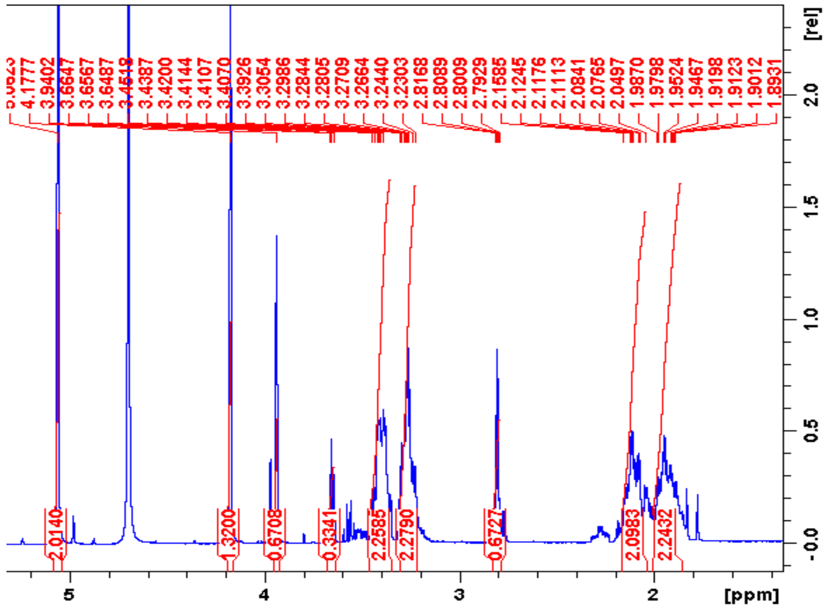

Figure S8. ^{1}H NMR spectrum of (*E*)- and (*Z*)-**3**.

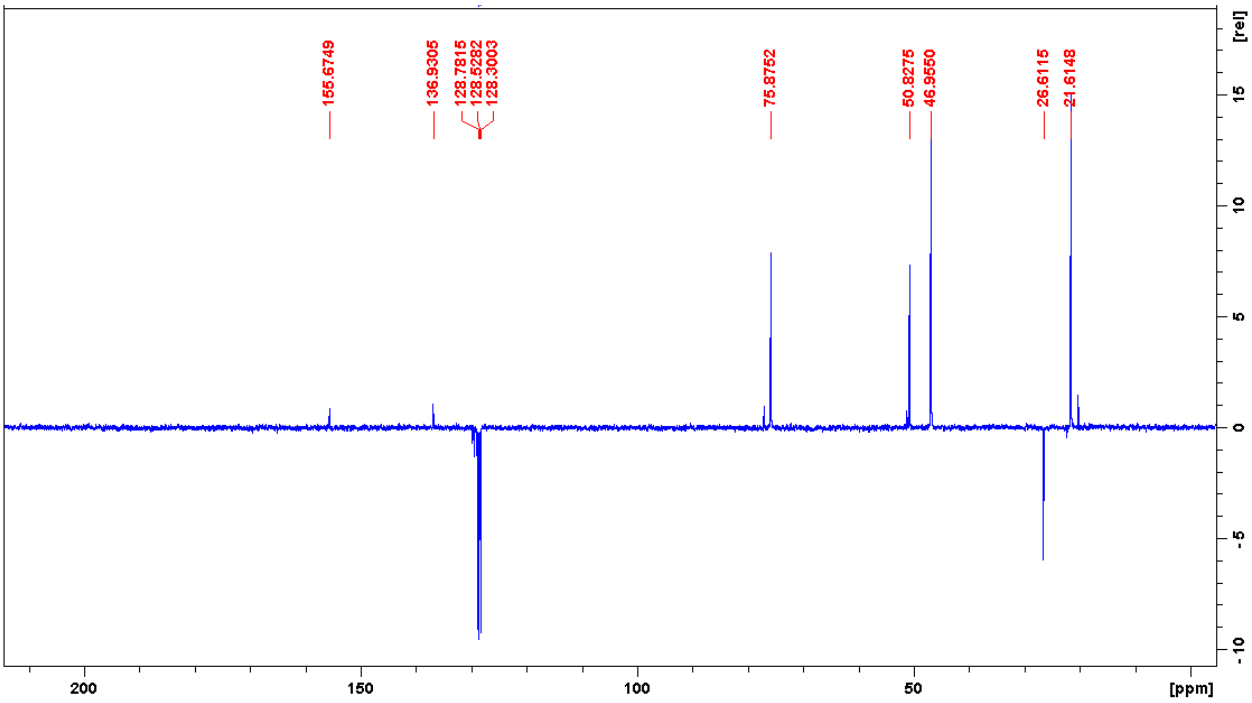

Figure S9. ^{13}C NMR spectrum of (*E*)-**3**.

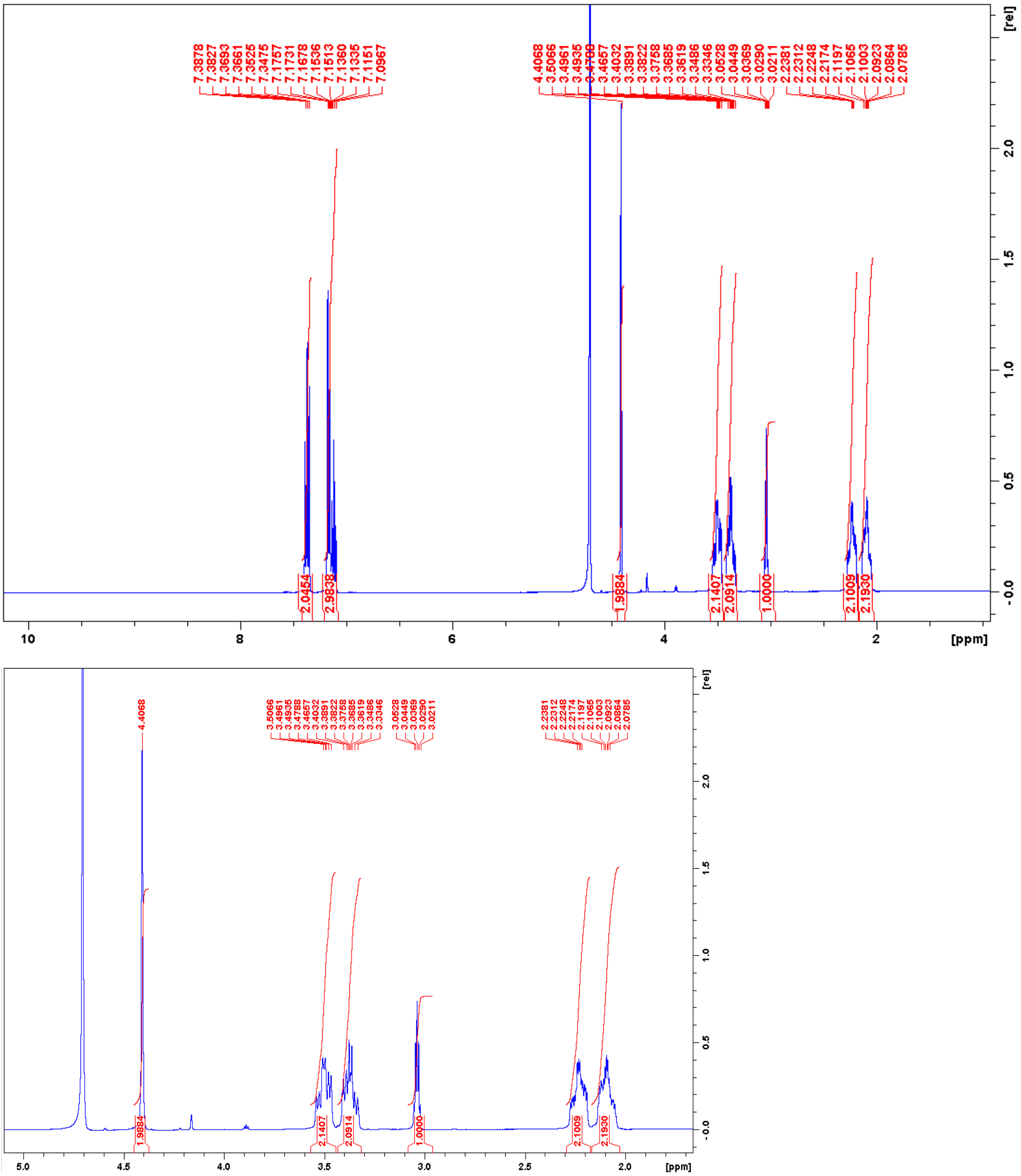

Figure S10. ^{1}H NMR spectrum of (*E*)-**4**.

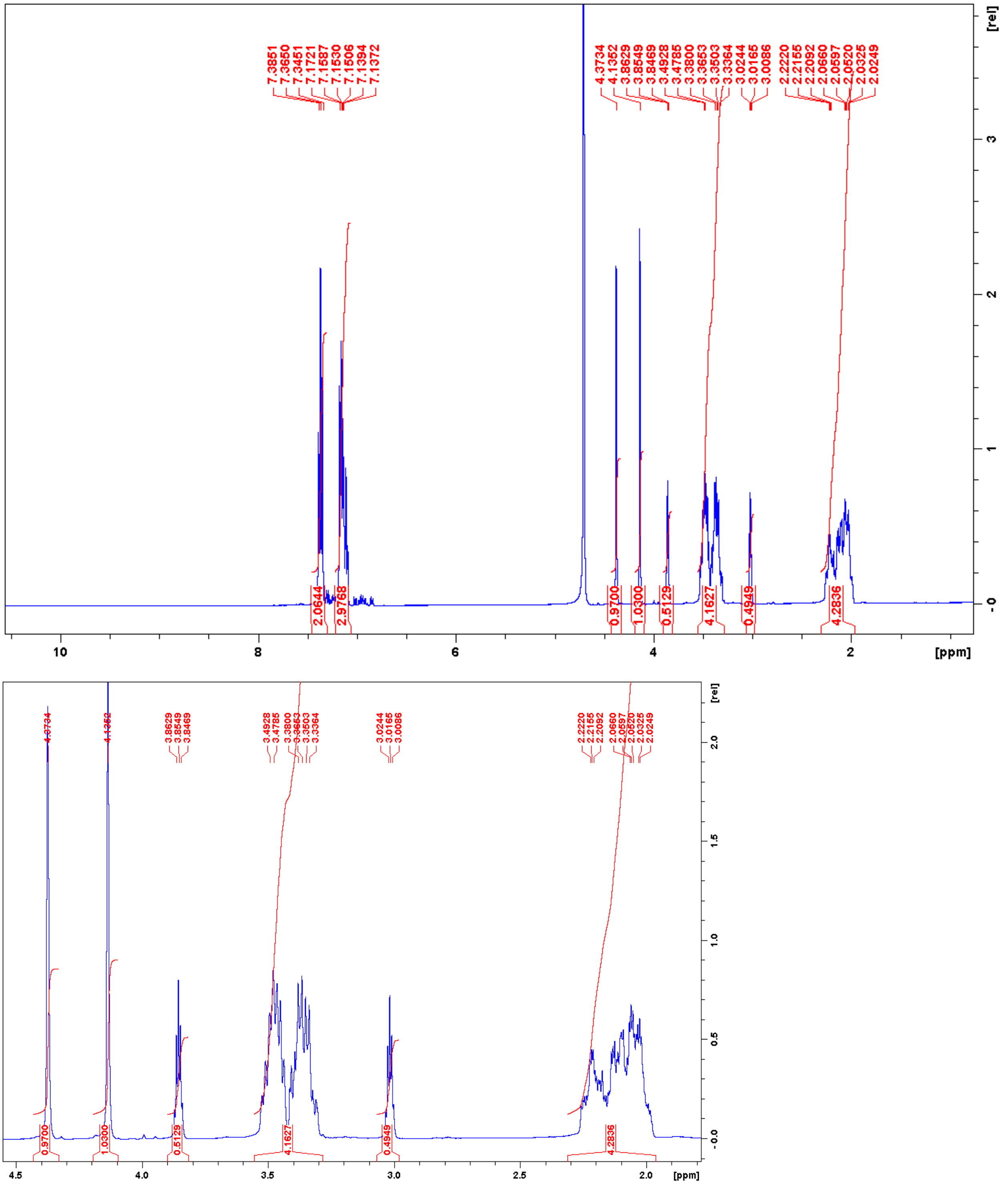

Figure S11. ^{1}H NMR spectrum of (*E*)- and (*Z*)-**4**.

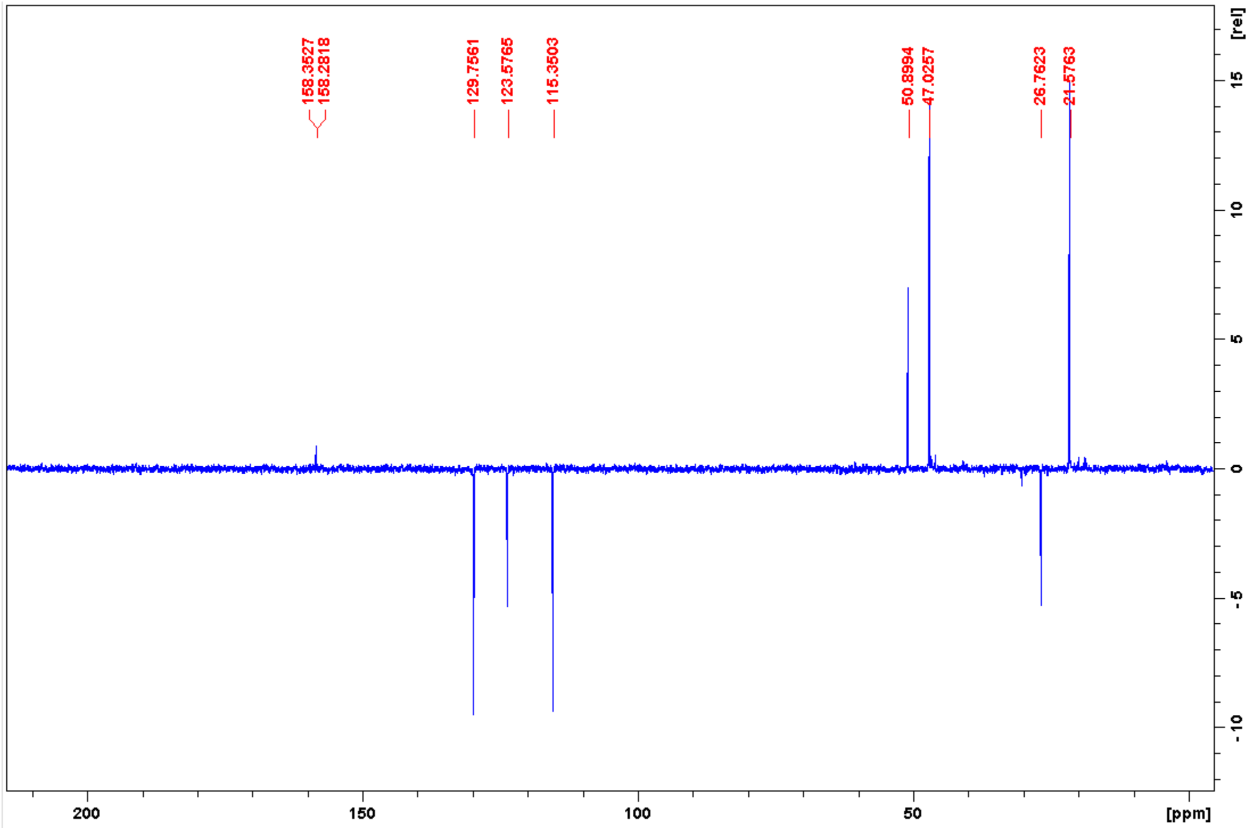

Figure S12. ^{13}C NMR spectrum of (*E*)-**4**.

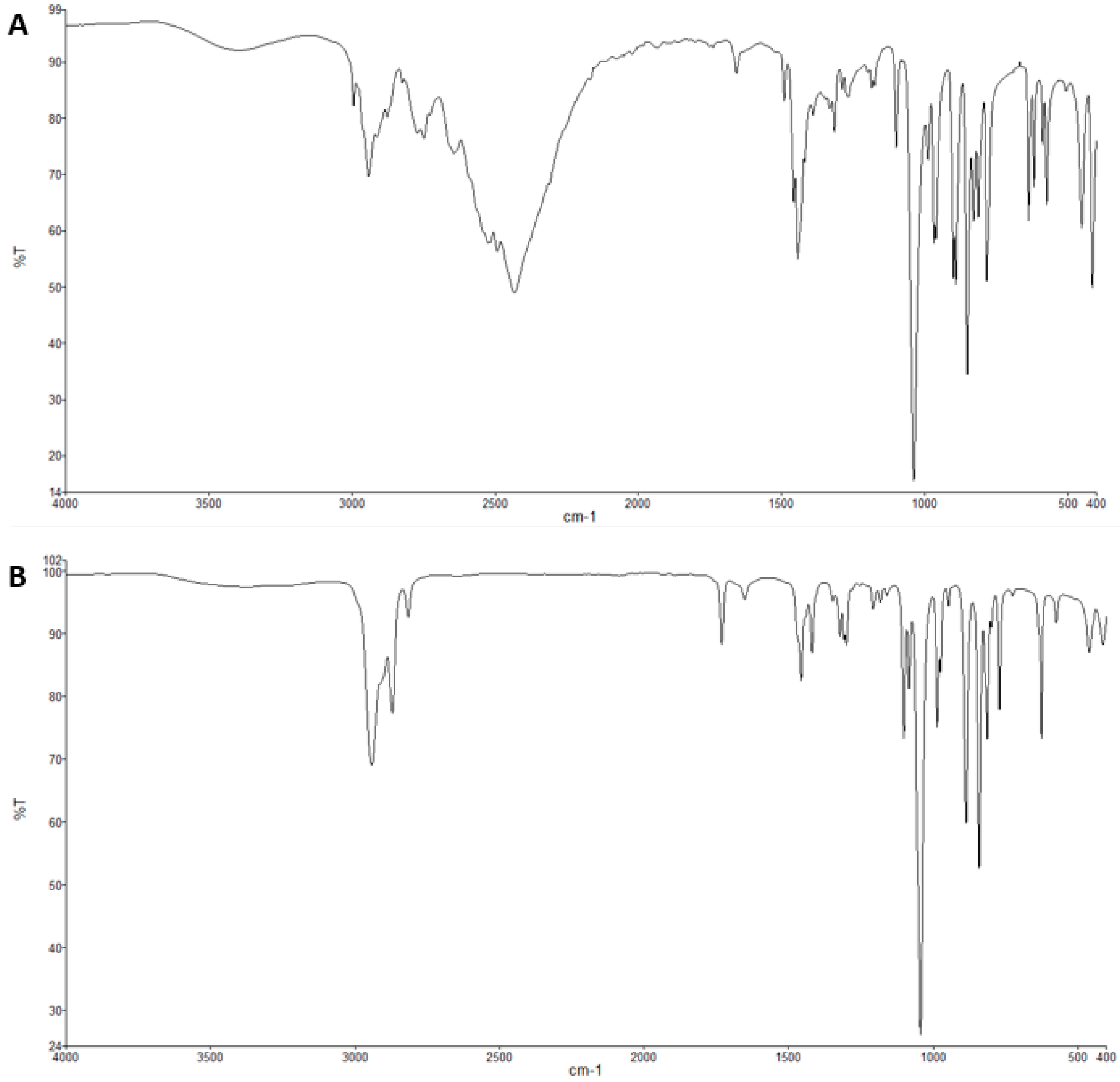

Figure S13. ATR spectrum of (**A**) (*E*)-**1**·HCl and (**B**) (*E*)-**1.**

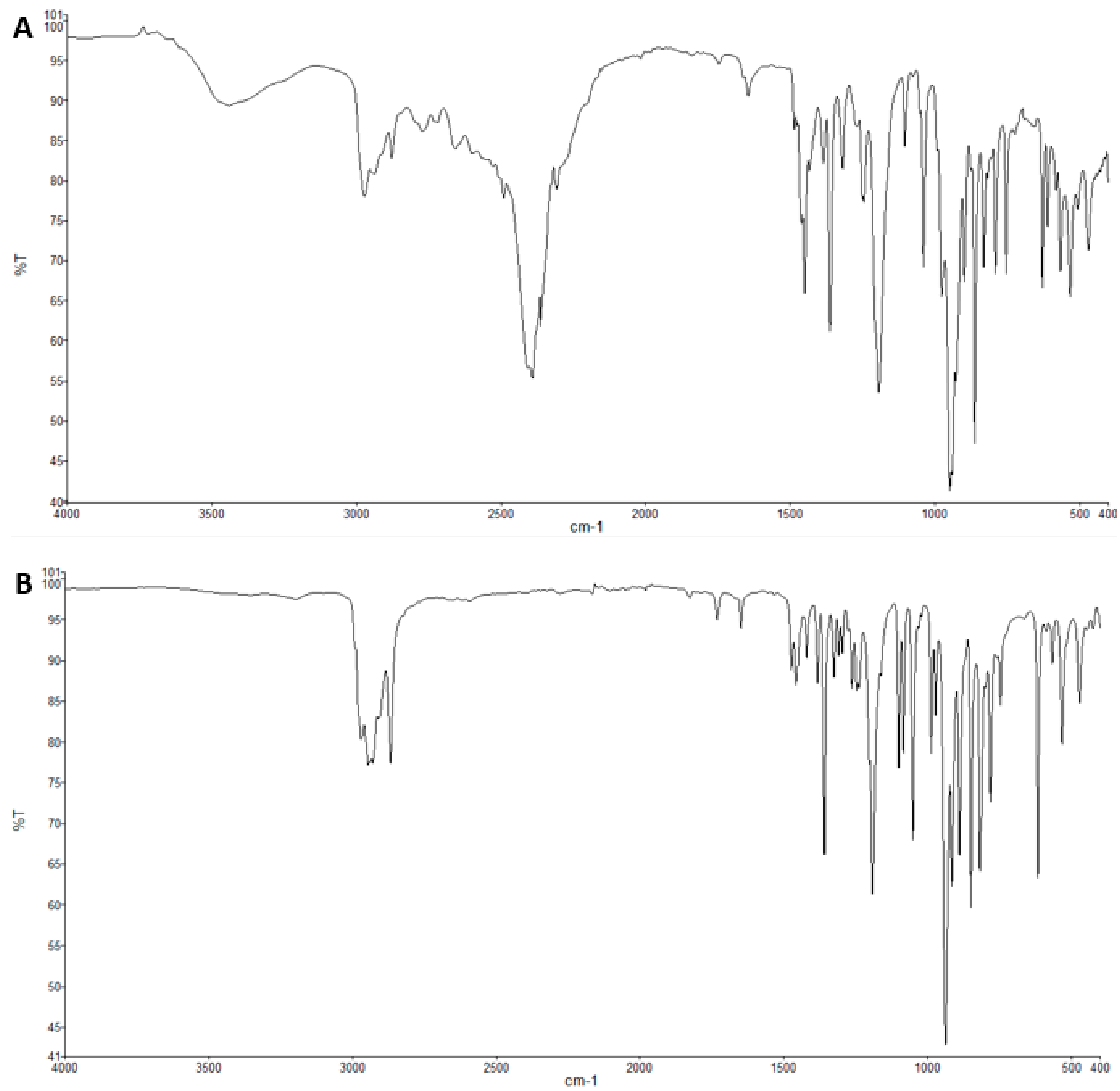

Figure S14. ATR spectrum of **A**) (*E*)-**2**·HCl and **B**) (*E*)-**2.**

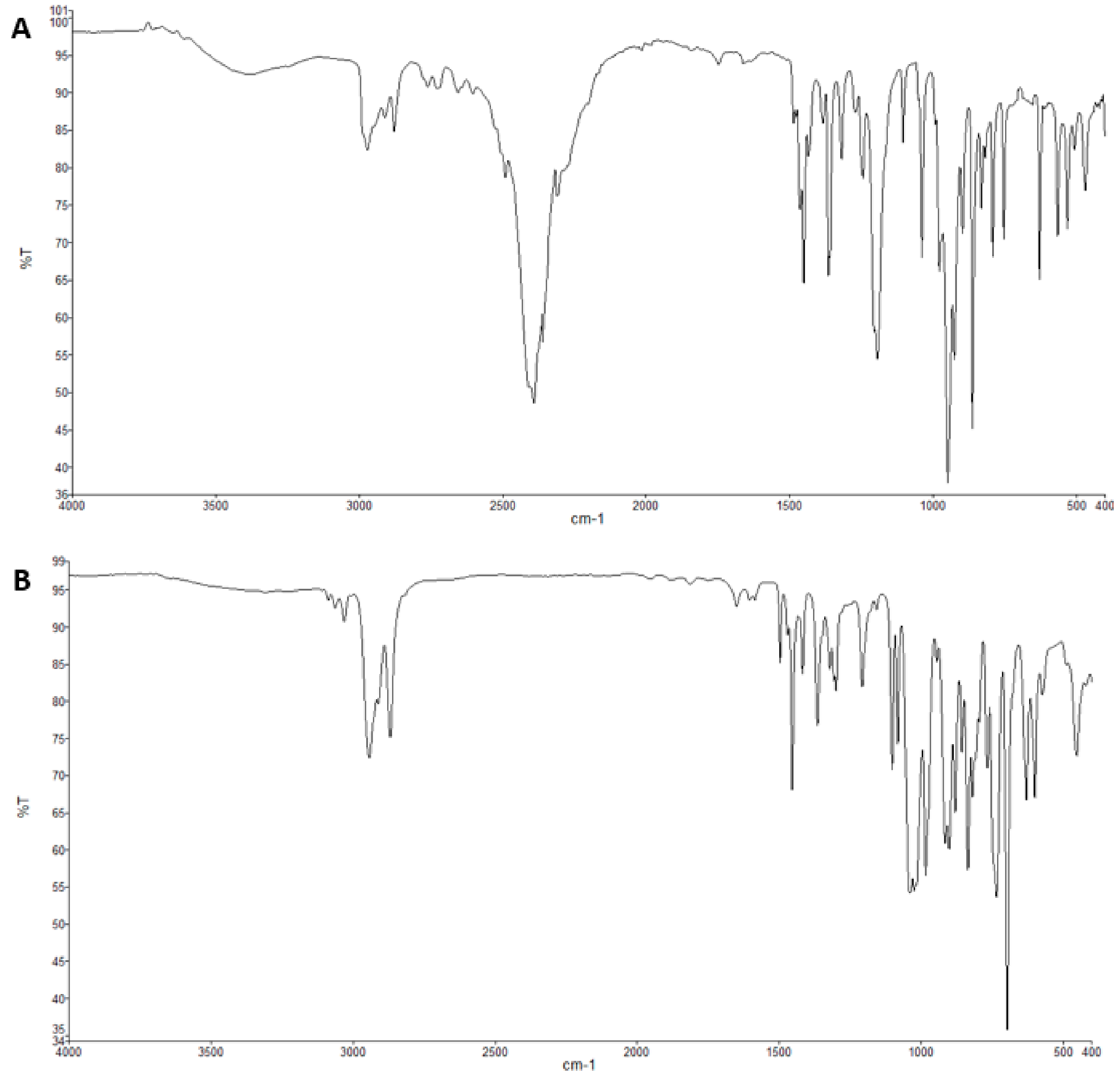

Figure S15. ATR spectrum of **A**) (*E*)-**3·**HCl and **B**) (*E*)-**3**.

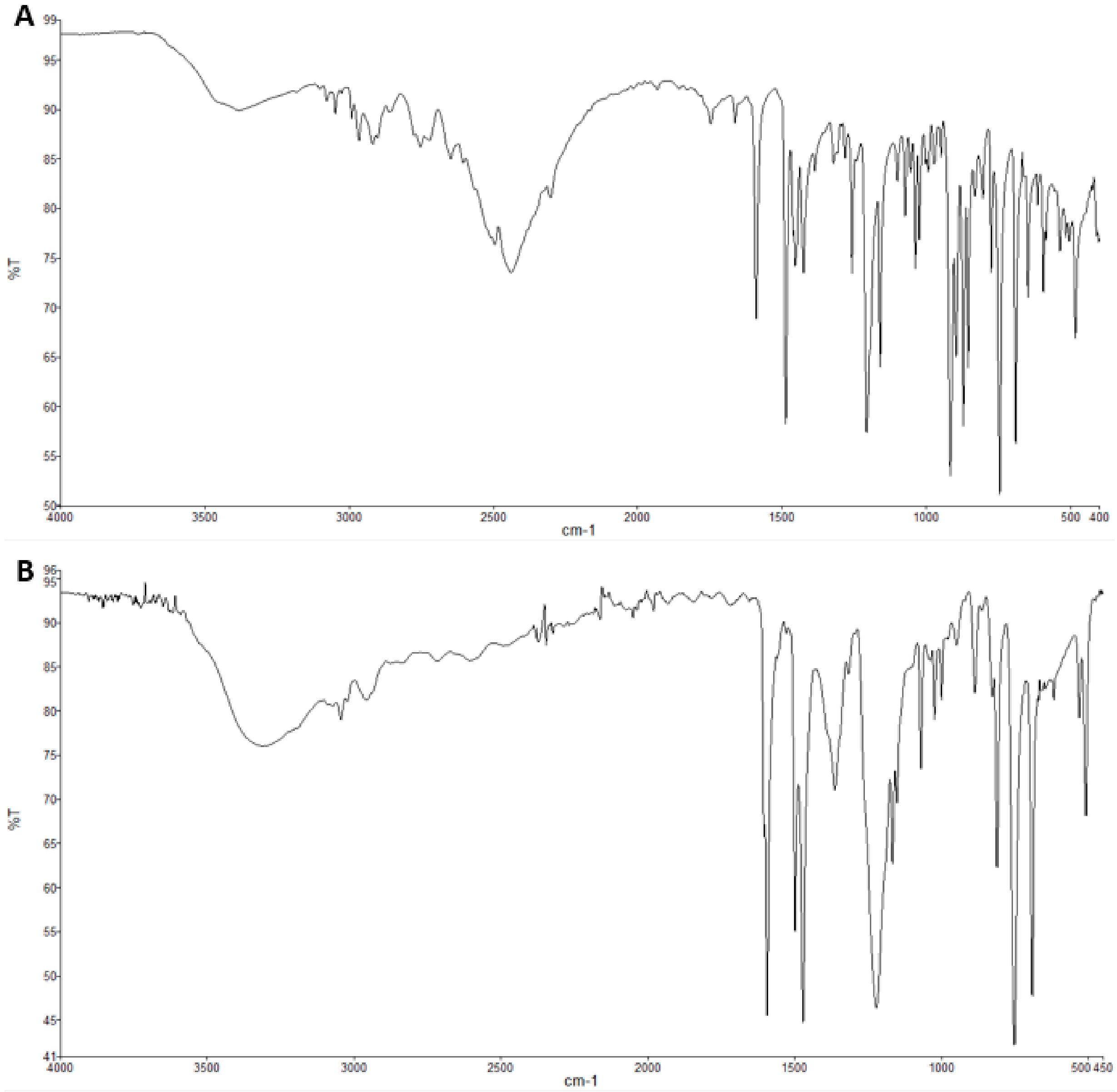

Figure S16. ATR spectrum of **A**) (*E*)-**4**·HCl and **B**) (*E*)-**4**

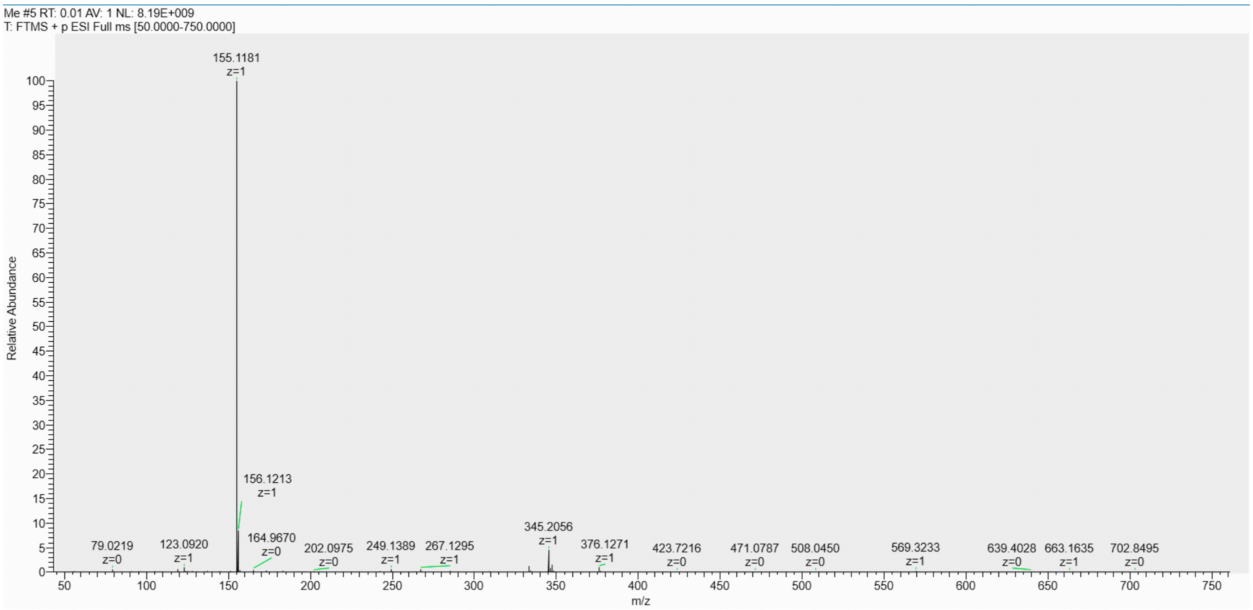

Figure S17. HRMS spectrum of (*E*)-**1**.

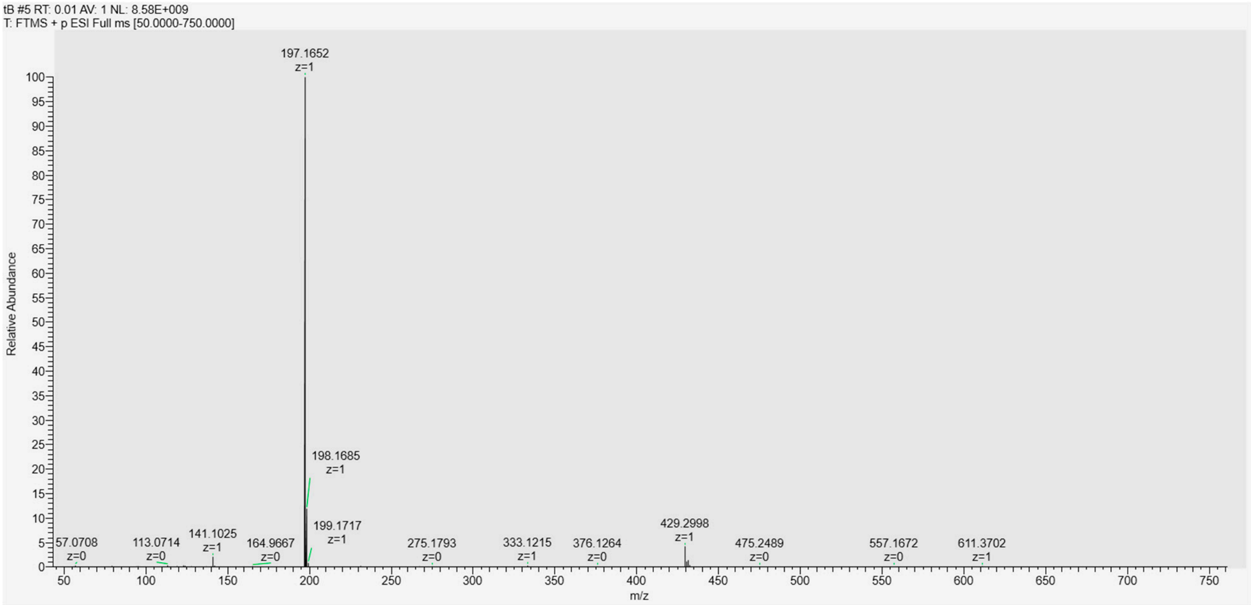

Figure S18. HRMS spectrum of (*E*)-**2**.

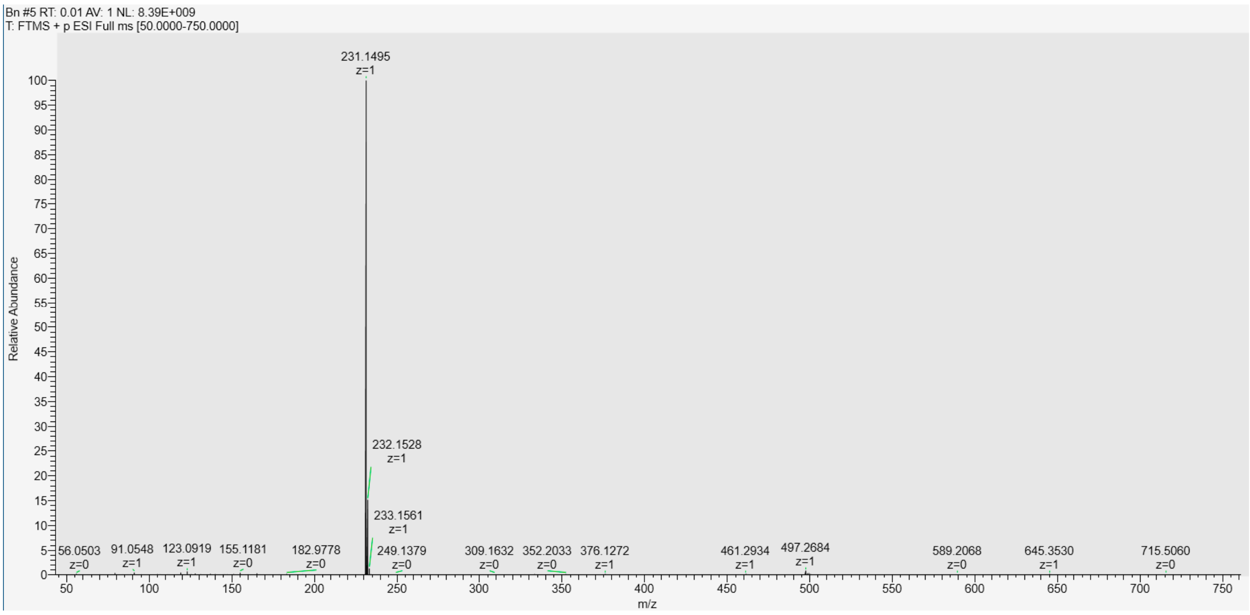

Figure S19. HRMS spectrum of (*E*)-**3**.

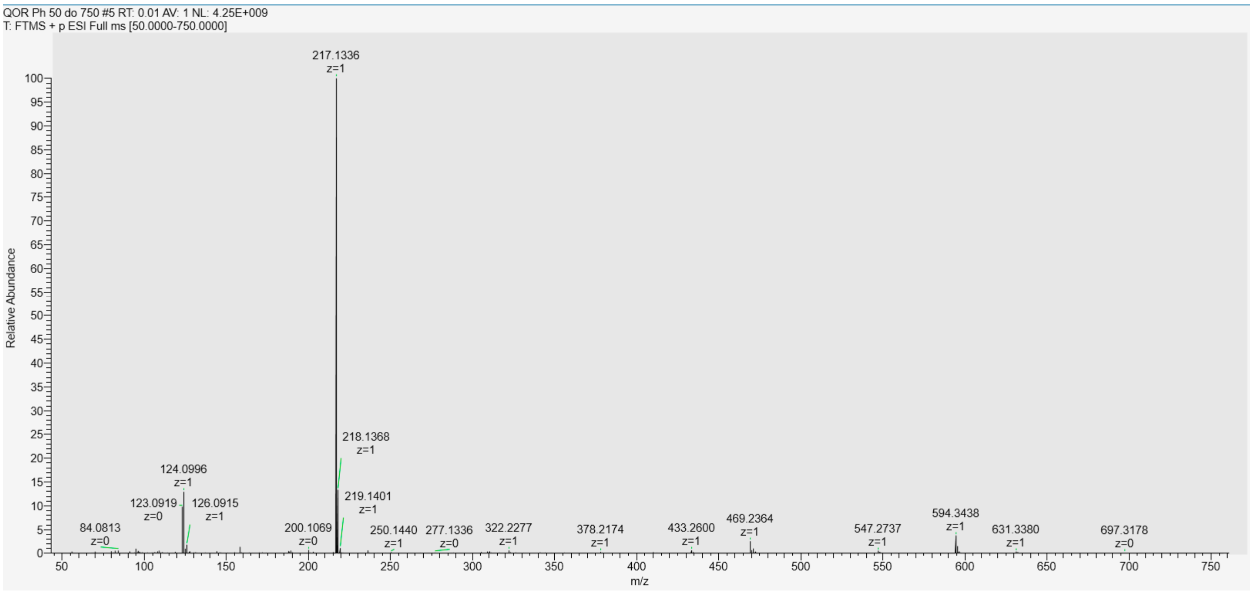

Figure S20. HRMS spectrum of (*E*)-**4**.

Conformer **RI1-I**

29
0.000000

C	-0.000000000000	-0.000000000000	1.544218482439
C	0.000000000000	0.000000000000	0.000000000000
N	1.445842459742	0.000000000000	-0.468284369464
C	2.208989502155	-1.116962029390	0.211193385353
C	2.401259961458	-0.731931773187	1.707882207289
C	1.402358082255	0.397767208133	2.042622145902
C	2.093871696472	1.346879288239	-0.170279489911
C	1.848843163828	1.680877634863	1.314426881731
N	2.115579033642	-1.899355168265	2.516756399193
H	-0.268601762371	-0.984182049505	1.926786106475
H	-0.740958798474	0.713172758686	1.906845628574
H	-0.464360035489	0.889128950595	-0.426943183012
H	-0.479068853614	-0.881734563549	-0.425102348115
H	3.167707230179	-1.224078678735	-0.291592448944
H	1.627955275382	-2.030077982854	0.099279712046
H	1.396636168584	0.561132143194	3.120874871747
H	1.653913243801	2.071934492481	-0.854682505936
H	3.154058073446	1.234881153380	-0.393686372452
H	2.763801493350	2.076454893033	1.749120632864
H	1.070510447557	2.439546442038	1.415906006113
O	3.107800458472	-2.873816424713	2.141400447581
C	2.591867490139	-4.197346016016	2.328310294173
H	3.387922253231	-4.865186474699	1.999497683600
H	1.690853744965	-4.352452696452	1.728045709770
H	2.369271432171	-4.384211505092	3.382882618298
H	1.467718003744	-0.157371694760	-1.476334104078
O	3.705800662056	-0.208842398914	1.916484940328
H	2.325156959146	-1.664685733924	3.489952714141
H	4.330197963815	-0.947133552170	1.938703073026

Conformer **RI1-II**

29

0.931530

C	0.000000000000	0.000000000000	1.541486478836
C	0.000000000000	0.000000000000	0.000000000000
N	1.444359552408	0.000000000000	-0.476246609900
C	2.100237849303	-1.327653464331	-0.163414539962
C	1.939248869973	-1.620274308911	1.358605347691
C	1.416757231504	-0.338228048233	2.043397040693
C	2.217292661935	1.136172865872	0.176406273133
C	2.366949818081	0.818658511714	1.681011219981
N	0.963414060222	-2.687239647051	1.521933268224
H	-0.732534189483	-0.721902046016	1.897501510966
H	-0.287322299183	0.981156214969	1.924512958361
H	-0.480176880034	0.878710299311	-0.430209059272
H	-0.459804640280	-0.898319985249	-0.410134706358
H	3.153504325839	-1.238798627822	-0.423640170603
H	1.630275518189	-2.094016774348	-0.775835801901
H	1.423715988842	-0.498902925065	3.121794526765
H	1.647851555199	2.046151324736	-0.014082903108
H	3.174881412567	1.210046439385	-0.338733035223
H	3.393870825654	0.542993037460	1.916450627627
H	2.116389578549	1.703388788945	2.267187508333
O	1.577884537940	-3.862588672402	0.963556645563
C	0.572508622629	-4.764130741558	0.486984156447
H	1.118934639874	-5.608914273934	0.068087998767
H	-0.044197243720	-4.290006814985	-0.282442802633
H	-0.063340997939	-5.110092261815	1.307200104054
H	1.454948988834	0.135525432087	-1.487873070657
O	3.195521639056	-1.919399720930	1.935945978337
H	0.882870421647	-2.882945969225	2.522951647158
H	3.433346884849	-2.827523558257	1.703030698206

Conformer **RI1-III**

29

1.105472

C	-0.000000000000	0.000000000000	1.544329075261
C	0.000000000000	0.000000000000	0.000000000000
N	1.446168701919	0.000000000000	-0.472515232783
C	2.211503295434	-1.126373122083	0.180521596400
C	2.382686646255	-0.803177023789	1.683182849947
C	1.413914680616	0.352528744295	2.043460241873
C	2.106094435072	1.336935328556	-0.155443988910
C	1.888387282396	1.638234037559	1.341058820470
N	2.045804020930	-2.003650741081	2.437373200941
H	-0.295370772087	-0.977410496896	1.923847264448
H	-0.721113547416	0.733292941229	1.907342396332
H	-0.463360596430	0.889036264140	-0.428111726888
H	-0.478111568568	-0.881821634073	-0.426389640930
H	3.186141578507	-1.201234661715	-0.299310679532
H	1.641473756902	-2.042572297006	0.024858612278
H	1.416567487854	0.484153642851	3.124740053517
H	1.657151990901	2.078038976392	-0.816424107050
H	3.161116847105	1.224886687650	-0.403038375713
H	2.821392819345	1.996696755324	1.770364216845
H	1.137568816113	2.419999275795	1.470182790148
O	2.419530861992	-1.745226850415	3.796575332652
C	1.373077072412	-2.156387228505	4.686063977346
H	1.747635776021	-1.938915690529	5.686297000338
H	1.177921732372	-3.227260931046	4.588575070262
H	0.453820799496	-1.593298647211	4.498677152904
H	1.463488385424	-0.140989930506	-1.483292665175
O	3.738663827195	-0.410183588373	1.840635661493
H	2.674785717957	-2.754922579515	2.146623007191
H	3.936898957034	-0.399357289670	2.787878550424

Conformer **RI1-IV**

29

1.464099

C	0.000000000000	-0.000000000000	1.541355466175
C	0.000000000000	0.000000000000	0.000000000000
N	1.446804071804	0.000000000000	-0.475914038478
C	2.104266028928	-1.323957932750	-0.164629888437
C	1.929772120547	-1.626856228509	1.342320186147
C	1.415477958623	-0.343000294515	2.041246526449
C	2.219159534417	1.136040870625	0.180142497850
C	2.369308640539	0.810843071532	1.682596714359
N	0.953571667090	-2.703205443718	1.464047681525
H	-0.727492927334	-0.725534307099	1.898024055551
H	-0.290099108719	0.980606867197	1.923876077143
H	-0.477049250723	0.879348574970	-0.432387411089
H	-0.459497257461	-0.897950721557	-0.411625439586
H	3.162981767512	-1.240591786106	-0.405249344154
H	1.641538472963	-2.082341993586	-0.796504035092
H	1.409885853730	-0.514710650915	3.116301611615
H	1.645976774049	2.044343052209	-0.006915956545
H	3.175515733204	1.214074290674	-0.336776511012
H	3.396153262777	0.527349063251	1.910487093465
H	2.128301770939	1.695815903896	2.272323845900
O	0.901254994918	-3.028950587684	2.860996289104
C	-0.385054537376	-3.557853793236	3.199104971916
H	-0.340065838440	-3.757135294894	4.269535152086
H	-0.579300777691	-4.490904262378	2.661465465084
H	-1.176843671194	-2.834911821247	2.983430042893
H	1.458530695341	0.136840746024	-1.487290178214
O	3.219804859131	-1.990131839169	1.804001368044
H	1.355425209766	-3.533770165720	1.019856650701
H	3.128192660428	-2.372941879183	2.687574200442

Conformer **RI2-I**

38
0.000000

C	-0.000000000000	0.000000000000	1.544160848843
C	0.000000000000	0.000000000000	0.000000000000
N	1.445649722160	0.000000000000	-0.467227956339
C	2.205816626410	-1.117553596941	0.215526208974
C	2.400409382481	-0.728984114231	1.709942537549
C	1.401521123606	0.401216396984	2.042151134868
C	2.093363961741	1.346933392577	-0.171299568479
C	1.845617784809	1.683948563944	1.312155469291
N	2.121458893871	-1.890837832892	2.526837990565
H	-0.264346552047	-0.985448706810	1.926535522895
H	-0.743770972225	0.710296676603	1.906806885330
H	-0.465001742610	0.888938954314	-0.426738045085
H	-0.478404609475	-0.882239572763	-0.424791283875
H	3.163918363373	-1.229780792208	-0.287077075218
H	1.621451429249	-2.027953018279	0.107792800232
H	1.394928759218	0.566142147694	3.120178594563
H	1.655219063761	2.070922639842	-0.858076993149
H	3.154005848144	1.233490627939	-0.391550354531
H	2.759572629094	2.081581390624	1.747078013411
H	1.066047410629	2.441844210690	1.410822345464
O	3.131320952432	-2.851371278325	2.169881144170
C	2.681174221478	-4.245285596503	2.325603121891
C	3.932353661309	-5.034589122353	1.952052790670
C	1.528415491891	-4.530315625758	1.364016928020
C	2.278284154183	-4.490271691704	3.779464114355
H	3.727352468511	-6.105302710712	2.013256164869
H	4.753615116122	-4.800729288249	2.632127480756
H	4.246122002715	-4.802932428144	0.931572170870
H	1.196576467300	-5.564926915379	1.473420270808
H	1.846400231544	-4.391037729968	0.326907559400
H	0.677417032476	-3.880156873260	1.577632409498
H	2.056471083663	-5.548664708236	3.931612908583
H	1.383691420300	-3.922745960627	4.045439125322
H	3.092157616255	-4.213545853662	4.454017774774
H	1.468218655886	-0.159469543120	-1.474792645468
O	3.706814243907	-0.203594448893	1.910812838354
H	4.321768635357	-0.949409076947	1.951577890437
H	2.320797234737	-1.640331205348	3.496868803040

Conformer **RI2-II**

38

0.313734

C	0.000000000000	0.000000000000	1.541608794768
C	0.000000000000	0.000000000000	0.000000000000
N	1.443664305116	0.000000000000	-0.476236031464
C	2.097685961194	-1.328638167023	-0.162239868097
C	1.935602859636	-1.621445539906	1.358398442303
C	1.416881099017	-0.338063259305	2.043114466916
C	2.217064054462	1.135625161310	0.175542348989
C	2.367265473583	0.818287279067	1.680136035856
N	0.960762964003	-2.686128916737	1.528601264238
H	-0.731176225985	-0.723235022471	1.897668219280
H	-0.288471141061	0.981032133900	1.924338440634
H	-0.480609700178	0.878862082546	-0.429582593876
H	-0.458733152204	-0.898131805721	-0.411439980111
H	3.151172801418	-1.241006171974	-0.421605787217
H	1.625366482849	-2.094271970421	-0.771981792793
H	1.423973813660	-0.498261795541	3.121608305333
H	1.648316877204	2.046173691654	-0.014663979799
H	3.174587457028	1.208632905515	-0.339841777431
H	3.394008787941	0.540995290960	1.914637338468
H	2.118418869729	1.703678488231	2.266163705989
O	1.579736106403	-3.861571162896	0.979527262268
C	0.606465444677	-4.848787819867	0.484433235069
C	1.507846537222	-5.987098531653	0.015419813259
C	-0.197939023596	-4.256647073775	-0.672366379404
C	-0.300303343477	-5.290326415843	1.633183586232
H	0.900631236617	-6.796479249784	-0.394914888947
H	2.093800502475	-6.382432373351	0.847224941815
H	2.193163102429	-5.642936976222	-0.762542751678
H	-0.910524588859	-4.993749096127	-1.048252819356
H	0.459174094135	-3.978665614472	-1.501404791479
H	-0.762487752553	-3.382353337067	-0.341959790296
H	-0.944275199324	-6.109611870359	1.306740880485
H	-0.946488105802	-4.475269510646	1.967451739564
H	0.296705231057	-5.643357318257	2.477428227945
H	1.453861149130	0.134315478610	-1.487919722551
O	3.195118861649	-1.923189979825	1.931617015613
H	3.411146353176	-2.840317624066	1.711718933977
H	0.884679683302	-2.870880121799	2.530770017387

Conformer **RI2-III**

38
2.115603

C	-0.000000000000	-0.000000000000	1.543599395965
C	0.000000000000	0.000000000000	0.000000000000
N	1.447081878392	-0.000000000000	-0.468790173400
C	2.213576736539	-1.115394639984	0.202786503715
C	2.393635560495	-0.761555425156	1.697037207784
C	1.405737810407	0.381799295417	2.043078566053
C	2.098734064207	1.344189794631	-0.163696068921
C	1.860227372542	1.665887117737	1.325035025840
N	2.100693724354	-1.954082103635	2.479518739086
H	-0.273473345155	-0.982265896224	1.924411713299
H	-0.738058304948	0.715998570017	1.907019528280
H	-0.462455111332	0.889199870231	-0.428867474764
H	-0.477838313405	-0.881749410706	-0.426989971232
H	3.185273703741	-1.201996550853	-0.280978338831
H	1.639942980548	-2.032815812712	0.069433594817
H	1.403948960440	0.521010842132	3.122412341796
H	1.656072088855	2.073579485535	-0.841776610042
H	3.157491188507	1.231457809506	-0.394072785758
H	2.781873002978	2.050203211763	1.756328211772
H	1.092112960738	2.434003789681	1.434144382611
O	2.526417734015	-1.641495161181	3.812688645761
C	1.736748331216	-2.319146120494	4.854513981493
C	2.393108842567	-1.809047834212	6.134485715678
C	1.898127094892	-3.831665617782	4.706180862799
C	0.271790138250	-1.896003218318	4.768733751690
H	1.902791720576	-2.251298901904	7.003987405762
H	2.307389997377	-0.722455581055	6.207072429708
H	3.450505287423	-2.079414086081	6.159892408070
H	1.398621472259	-4.344197211011	5.531159586437
H	2.955064141244	-4.108079194525	4.723125464557
H	1.449597114265	-4.187798644368	3.775699850083
H	-0.288479807044	-2.334045057644	5.597533315670
H	-0.180405464880	-2.241706776619	3.838283086383
H	0.177217920597	-0.809259825126	4.835007453941
H	1.469395336305	-0.152766565026	-1.477586710523
O	3.743748584273	-0.333083086067	1.830992624194
H	3.955326688681	-0.339751541474	2.775814116104
H	2.739825481391	-2.691342404467	2.173633736301

Conformer **RI2-IV**

38
9.148380

C	0.000000000000	0.000000000000	1.542180316273
C	0.000000000000	0.000000000000	0.000000000000
N	1.444265870566	0.000000000000	-0.480454332607
C	2.125598301849	-1.298964199781	-0.109058496979
C	2.069531727191	-1.486255051358	1.433220133124
C	1.438673648583	-0.212621051275	2.048144833789
C	2.199321772571	1.184577815390	0.101146865593
C	2.289617901220	1.004921691828	1.634025349964
N	1.419846353255	-2.764450642403	1.744787845553
H	-0.664708081684	-0.783670225710	1.897080818063
H	-0.367815648633	0.954830315636	1.922478415032
H	-0.475467772395	0.880030795097	-0.433188162737
H	-0.458585922533	-0.901556036404	-0.401501231845
H	3.162464849356	-1.228960826139	-0.435017324409
H	1.614190958008	-2.101305140482	-0.633230770270
H	1.455082074937	-0.323912540618	3.133152242525
H	1.647597662759	2.077402103839	-0.193625026422
H	3.176936673973	1.204391169915	-0.380495646354
H	3.324454063375	0.861076026983	1.938950207584
H	1.915417007152	1.900585522317	2.131801471915
O	0.142248556507	-2.853350291186	1.110174965203
C	-0.190456117023	-4.234581034233	0.703627424227
C	-1.590969886426	-4.067183257053	0.121463378693
C	-0.203511472988	-5.135766052763	1.937430158903
C	0.800416551656	-4.731120461056	-0.347252972497
H	-1.966739711640	-5.030880225623	-0.227593795430
H	-1.579562905891	-3.381360145292	-0.730037575471
H	-2.280379384614	-3.680920045364	0.874815703913
H	-0.565724411494	-6.130501012017	1.669452364372
H	-0.867389285131	-4.727466098352	2.703463448436
H	0.798834254876	-5.249694042827	2.355352110470
H	0.557851741656	-5.757067602835	-0.631848846584
H	1.819074664041	-4.721563691373	0.043707792646
H	0.753604254008	-4.119884177085	-1.253609201467
H	1.445856378238	0.081673580800	-1.497849042051
O	3.364748445487	-1.583190490194	1.986554393845
H	3.681123918841	-2.487398927300	1.854349658132
H	1.241104944383	-2.787184352353	2.749266645377

Conformer **RI3-I**

37
0.000000

H	0.000000000000	0.000000000000	4.537608134372
C	0.000000000000	0.000000000000	0.000000000000
C	0.328295218506	0.000000000000	-1.344336403198
C	1.166185688417	-0.998137335565	-1.860778965389
C	1.680901080754	-1.995848761667	-1.033563268146
C	1.358260606066	-1.998709809931	0.318181653743
C	-3.644591803599	-0.726408538075	3.634876155396
C	-3.897230890896	-0.205919960381	5.063528559695
N	-3.369141412453	-1.242355961493	6.047729335022
C	-1.858969978935	-1.292654541565	6.007382328497
C	-1.398346685521	-1.482585091291	4.539552309497
C	-2.640924605219	-1.891798784801	3.701977364359
C	-3.938032782857	-2.615580896724	5.721839156799
C	-3.298479997416	-3.094588095794	4.401152087570
N	-0.908040449492	-0.165276507677	4.098972628660
H	-3.258444543670	0.085091178408	3.020481289977
H	-4.575997600721	-1.075697279323	3.184138348270
H	-4.953445097989	-0.061771472239	5.290555699552
H	-3.352899458540	0.715639081554	5.264951875209
H	-1.532079806058	-2.145944602880	6.599418853052
H	-1.478546459966	-0.370148112245	6.446120696210
H	-2.311924061125	-2.177554494679	2.704530240541
H	-5.019237984699	-2.492569019919	5.654240175410
H	-3.712193646093	-3.266971693093	6.566025122415
H	-2.547137683856	-3.860617775900	4.591933148227
H	-4.067508412110	-3.530416967559	3.762556373414
O	-0.599011724842	-0.173096794650	2.665621691659
C	0.246634275300	-1.108339206503	2.271171239812
C	0.511725202945	-1.006582436067	0.852850812878
H	-0.640148275966	0.772757124529	0.407211990228
H	-0.057229933778	0.771725481363	-2.000023654599
H	1.419800997823	-0.990122440619	-2.914885941464
H	2.331746182286	-2.759474610837	-1.441608866276
H	1.752662608614	-2.758298794246	0.982843669254
H	-3.661474664594	-0.978784849936	6.989189025283
O	-0.412451498251	-2.480188917537	4.558730579214
H	0.118810721761	-2.327922610056	3.727711935112

Conformer **RI3-II**

37
0.443985

H	-0.000000000000	-0.000000000000	4.537928360559
C	0.000000000000	0.000000000000	0.000000000000
C	0.331530295767	0.000000000000	-1.343639813670
C	1.154576518714	1.009296931098	-1.862185020701
C	1.651955820103	2.018144626104	-1.037659626522
C	1.326596896346	2.020946495503	0.313377006288
C	-3.188739542395	0.394528990088	6.027443947087
C	-4.351427858602	1.167863863353	5.369687848217
N	-3.754906830108	2.320795114000	4.579442408443
C	-2.662511090132	1.807773161111	3.667826033483
C	-1.426402738972	1.444174964816	4.550424113127
C	-1.929082409193	1.279515165419	6.006912364148
C	-3.205086282456	3.378908661894	5.530049426960
C	-2.284235088290	2.678289957372	6.547844106744
N	-0.911383145038	0.150886680860	4.103138610568
H	-2.998527774480	-0.537592575131	5.496308310999
H	-3.455544988059	0.145153577214	7.055032648070
H	-5.035045123315	1.610062393202	6.094758362425
H	-4.927182296582	0.559125315192	4.672724017329
H	-2.407239164195	2.600489081243	2.967541599525
H	-3.057896035198	0.946835120138	3.131041224373
H	-1.134149242023	0.835480484110	6.607004289667
H	-4.062335234755	3.871570609200	5.988582479859
H	-2.659280058560	4.092118702308	4.913958601367
H	-1.387660557028	3.276815028538	6.689659899100
H	-2.782581640643	2.574615176513	7.513694773122
O	-0.611856570635	0.176420343790	2.662163135451
C	0.226504985613	1.117677679360	2.268210661589
C	0.493597338472	1.018227533920	0.849822521357
H	-0.626978159538	-0.782275394500	0.409631560676
H	-0.038949209132	-0.781257192735	-1.996704827214
H	1.411625698854	1.000546274931	-2.915432918116
H	2.293047124463	2.789278294387	-1.447042538589
H	1.710427755551	2.787375635160	0.976478325234
H	-4.485246736085	2.744151527469	4.006399982026
O	-0.477255980224	2.482184047154	4.532665625380
H	0.071437384473	2.319101279175	3.714219642392

Conformer **RI3-IV**

37

1.675567

H	0.000000000000	0.000000000000	4.528306869414
C	0.000000000000	0.000000000000	0.000000000000
C	0.337844153495	0.000000000000	-1.341954576732
C	1.249624538796	0.943986728751	-1.834670401313
C	1.830177639193	1.885805384752	-0.985754858998
C	1.500544538945	1.886335067532	0.364266908925
C	-2.731904655831	0.124671405931	6.279201674554
C	-3.903727069508	0.219442544678	5.283357268324
N	-3.907672708765	1.618421697636	4.686595378924
C	-2.717519693207	1.800129916171	3.770333508907
C	-1.410980263363	1.464762361394	4.557202143217
C	-1.786486691534	1.319598730052	6.054919863878
C	-3.885205087152	2.666088032228	5.789462957286
C	-2.503738531125	2.614921887601	6.478478795847
N	-0.913550034921	0.162528351141	4.101409770505
H	-2.218154626920	-0.823453800769	6.134023227711
H	-3.100008278226	0.150466915288	7.306959852118
H	-4.877447864793	0.065321550668	5.748217489672
H	-3.789212805120	-0.471333626707	4.448698562887
H	-2.696324590782	2.843020508748	3.460436038017
H	-2.858795037395	1.156541349330	2.903839910470
H	-0.863217349372	1.204941594651	6.623206331402
H	-4.707971223740	2.422789903298	6.462232269724
H	-4.089985955825	3.628782088569	5.321316484720
H	-1.900292134954	3.477822080252	6.199894311240
H	-2.635339789082	2.633130820518	7.560889560524
O	-0.624949485697	0.187532144349	2.662644825529
C	0.313603074365	1.043529735839	2.292954069501
C	0.577809832303	0.950717930583	0.874556311572
H	-0.695009900374	-0.733547208060	0.389594355173
H	-0.096117068308	-0.730985823553	-2.013861590126
H	1.510505132928	0.936022912594	-2.886996689847
H	2.538760092722	2.606431532322	-1.375385070692
H	1.947277807082	2.600279645616	1.046166143508
H	-4.760175289052	1.738135555298	4.138420564952
O	-0.488769174295	2.514222135222	4.430792375980
H	0.109394690651	2.272787229586	3.666522086932

Conformer **RI4-I**

36

0.000000

C	0.000000000000	-0.000000000000	1.544256216812
C	0.000000000000	0.000000000000	0.000000000000
N	1.445712502729	0.000000000000	-0.468563262831
C	2.203685243091	-1.123698333792	0.201599815387
C	2.393595902202	-0.754081566781	1.704054260346
C	1.405647045110	0.382951667467	2.045245199974
C	2.098205268035	1.342733587811	-0.163581102154
C	1.863272855790	1.666636442282	1.325373177882
N	2.079259326578	-1.925458284290	2.499952282434
H	-0.279799471602	-0.981622412037	1.924818068405
H	-0.733530334643	0.720368267024	1.907674771095
H	-0.464690453919	0.888911341366	-0.426999439933
H	-0.477605743937	-0.882888439870	-0.424214702381
H	3.164913669068	-1.225743237396	-0.298175336800
H	1.623906534380	-2.036458176770	0.072895287882
H	1.400956848469	0.539029483850	3.124707530701
H	1.655720913360	2.074231243399	-0.839428376998
H	3.156618016774	1.230400930155	-0.395301232401
H	2.784123569029	2.048597025520	1.759904906101
H	1.093783091496	2.432905354848	1.436328328116
O	3.024980635460	-2.935960016794	2.121334984918
C	2.441273728795	-4.110821619074	1.635419259523
C	1.082021486547	-4.393428754491	1.716737767863
C	0.618915554608	-5.604966164382	1.196072884777
C	1.497345420228	-6.511901303264	0.610636044137
C	2.858645466532	-6.208783970844	0.547626422231
C	3.338572943827	-5.006212706087	1.057178373109
H	0.407829702572	-3.696888362738	2.193729725238
H	-0.437145029818	-5.839178744865	1.264578081123
H	1.129342635492	-7.450411232696	0.214898918093
H	3.552822270610	-6.911547953008	0.102218659953
H	4.393199526963	-4.760315167166	1.023498568815
H	1.466016878392	-0.152493258255	-1.477514912087
O	3.701744192924	-0.251670887093	1.927325418631
H	4.321052759757	-0.994278507015	1.923078661382
H	2.300688391201	-1.734783734288	3.476780543407

Conformer **RI4-II**

36

0.971952

C	-0.000000000000	-0.000000000000	1.541314165507
C	0.000000000000	0.000000000000	0.000000000000
N	1.446086441826	0.000000000000	-0.471956682442
C	2.089735823444	-1.336982981929	-0.177729714413
C	1.897020966215	-1.663792853785	1.334863585414
C	1.407183903425	-0.380901974981	2.042782142278
C	2.224718304357	1.117780773058	0.203969988618
C	2.385051569877	0.758525565972	1.697965875640
N	0.874189673359	-2.692474657143	1.463139956970
H	-0.754875065008	-0.698491914734	1.897244172954
H	-0.257250672546	0.989157285105	1.925415213872
H	-0.478958052602	0.879742982896	-0.429383437164
H	-0.456491478451	-0.899177653682	-0.412816481189
H	3.149705707453	-1.244845856849	-0.407902363870
H	1.632169423604	-2.079913727115	-0.828031796181
H	1.407945726863	-0.559925385541	3.118325736724
H	1.654054514235	2.032773159597	0.043597225728
H	3.178685138410	1.206083650034	-0.315599253051
H	3.406857853656	0.449047599642	1.913850602380
H	2.166846589693	1.635289516789	2.308514228021
O	1.395016247902	-3.876614060123	0.844194599896
C	0.602835422663	-4.357180566744	-0.202454305048
C	-0.722557799366	-3.983658306202	-0.403063155559
C	-1.419988444890	-4.540215966160	-1.478289436964
C	-0.804845692263	-5.453257405483	-2.330390307820
C	0.522729830424	-5.820066363226	-2.103943337421
C	1.235444118034	-5.272205274941	-1.041270321835
H	-1.203650794185	-3.296528784463	0.278212162588
H	-2.456456700959	-4.264649897177	-1.635407279552
H	-1.355241417089	-5.883509676565	-3.157897385165
H	1.008260637084	-6.536560678295	-2.755848318955
H	2.264364824252	-5.551516827179	-0.848556683182
H	1.460488593780	0.150031803459	-1.481513030022
O	3.132532537972	-2.023435371878	1.918972385785
H	3.357827067681	-2.921911807655	1.641531471858
H	0.787548791492	-2.950185471911	2.446055654142

Conformer **RI4-III**

36

0.584433

C	-0.000000000000	0.000000000000	1.539769332093
C	0.000000000000	0.000000000000	0.000000000000
N	1.448523302990	-0.000000000000	-0.475342798833
C	2.100313307910	-1.329681039831	-0.180205853951
C	1.914005791994	-1.653112621794	1.321188703523
C	1.409440806162	-0.373894155116	2.036498515459
C	2.224627587065	1.123519317249	0.198328116030
C	2.379843918054	0.771325715814	1.694375701600
N	0.918892546532	-2.718474657587	1.417268486991
H	-0.746844410135	-0.703691252694	1.901442158527
H	-0.262315356394	0.988475846041	1.922431483014
H	-0.476943318918	0.878936197183	-0.433306334750
H	-0.458632461644	-0.898712244464	-0.411183174902
H	3.160897939773	-1.246739260861	-0.412512563900
H	1.640081102041	-2.078168766681	-0.825712127396
H	1.397764540058	-0.558688672421	3.109944518484
H	1.651030369222	2.035194757267	0.030365139605
H	3.179247502308	1.210575214757	-0.320355374734
H	3.404032711565	0.470053606409	1.911555261947
H	2.154120626052	1.649787074993	2.299547383349
O	0.835728210873	-3.072080384028	2.804502187466
C	-0.454439029185	-2.980854604650	3.332821282946
C	-1.601925596447	-3.196023940440	2.575952281254
C	-2.844320748575	-3.121287022663	3.206768585536
C	-2.933961884751	-2.847636663224	4.570372317715
C	-1.769988187616	-2.647331999862	5.312112271811
C	-0.522129931604	-2.707141470454	4.695335550874
H	-1.525145264361	-3.420803409953	1.520861481836
H	-3.744177653988	-3.292147564845	2.627368140788
H	-3.902258066209	-2.797742634896	5.052951606854
H	-1.829584166880	-2.440311352568	6.374032338576
H	0.392076829985	-2.557286291337	5.257245699707
H	1.462748057301	0.150419671947	-1.484679689313
O	3.193714127685	-2.042444738458	1.784821839968
H	3.092721942472	-2.424274020119	2.667712978991
H	1.303605777837	-3.557798771647	0.981537739722

Conformer **RI4-IV**

36

0.808412

C	-0.000000000000	0.000000000000	1.544778218616
C	0.000000000000	0.000000000000	0.000000000000
N	1.444299683339	-0.000000000000	-0.480992215218
C	2.200505681932	-1.163301902547	0.113166743638
C	2.338210437824	-0.944635947679	1.638819086863
C	1.433780670092	0.246527728077	2.047152548818
C	2.128379514628	1.311561266505	-0.118230633088
C	1.982412930922	1.532029913650	1.402023739535
N	1.892204370956	-2.170593542811	2.295672360526
H	-0.372318702811	-0.947772485829	1.929249384666
H	-0.659242315894	0.787376827731	1.911899395228
H	-0.465644825250	0.887384593110	-0.429121824127
H	-0.476016044073	-0.883254486502	-0.426015581861
H	3.185521674244	-1.202171370374	-0.349513457778
H	1.639522441174	-2.069274306606	-0.117276012660
H	1.438154547491	0.336675893466	3.132692307146
H	1.655060235745	2.090639233063	-0.715520104866
H	3.169109211250	1.210376863731	-0.425040545364
H	2.951895555649	1.793080676087	1.820863786274
H	1.296576641856	2.356507484044	1.603612203088
O	2.171842383691	-2.012824148442	3.692075079955
C	1.027796084420	-2.029308172784	4.496919817562
C	-0.136455353672	-2.708870103745	4.154982081036
C	-1.207454969352	-2.693336313724	5.049498186419
C	-1.110156499862	-2.018871092767	6.264981235158
C	0.071158528276	-1.353272882017	6.591764756606
C	1.146336287233	-1.350233591523	5.705979480277
H	-0.199087865419	-3.244451519553	3.217879248963
H	-2.117109183423	-3.225122101392	4.795617459721
H	-1.944728689343	-2.017397776049	6.955260673150
H	0.159192756107	-0.831895001845	7.537567806979
H	2.073074829322	-0.842852275603	5.946543624684
H	1.450564641160	-0.098919294160	-1.496998767671
O	3.709077955679	-0.657859663660	1.866511750014
H	3.869042965773	-0.698475355113	2.819884324292
H	2.509526369675	-2.935348951597	2.021487669439
